# Supplementary material for: Cell-cycle dependent DNA repair and replication unifies patterns of chromosome instability
Source: Nat Commun. 2025 Mar 28;16:3033. doi: 10.1038/s41467-025-58245-z (PMC11953314; doi:10.1038/s41467-025-58245-z)
Supplement: Supplementary file 1 — Supplementary Information [file 41467_2025_58245_MOESM1_ESM.pdf]

Supplementary document for Cell-cycle dependent DNA repair and replication unifies patterns of chromosome instability

Supplementary Tables

Supplementary Table 1: The major input parameters of the stochastic cell-cycle model. The top three parameters are estimated in the inference, while the other parameters are fixed.

parameter	symbol	default value	values used in main figures
double-strand break (DSB) rate per cycle	r	5	10 (Fig. 2f-g); {10, 30} (Fig. 3,5)
fraction of unrepaired DSBs per cycle	f_u	0	0.5 (Fig. 2d-e, 4); 1 (Fig. 2b-c); {0, 0.1, 0.3} (Fig. 3); {0.1, 0.3, 0.5} (Fig. 5)
probability of whole genome doubling (WGD) per cell	p_w	0	{0.1, 0.3, 0.5} (Fig. 5)
population size (number of cells)	N	2	3 (Fig. 1c); 10 (Fig. 2f, 5); 100 (Fig. 2b-e); 200 (Fig. 2g); {2, 3} (Fig. 3); {1000, 3000, 5000} (Fig. 4); number of clones (Fig. 6-8)
birth rate per cycle	b	1	
death rate per cycle	d	0	
number of DSBs per cycle	n	0	1 (Fig. 2b-c); 2 (Fig. 2d-e, 4); 5 (Fig. 1c)
maximum identifier (ID) of cycles with DSBs	n_d	0	1 (Fig. 1c); N (Fig. 2f-g, 5-8); {0, 1} (Fig. 3)
mode of repairing	m_p	1 (distance-based)	
probability of correct DSB repair	p_r	0	0.5 (Fig. 2d-e, 4)
mean number of DSBs during local fragmentation	n_l	0	50 (Fig. 2d-e); {0, 10, 30} (Fig. 3)
probability of DSB on a chromosome c	p_c	$\frac{1}{22}$	biased towards chr1 (Fig. 1c, 3)
model of evolution	m	0 (neutral)	1 (Fig. 6-8)
strength of selection	S	1	5 (Fig. 2b-e, 4)

Supplementary Table 2: The total copy numbers of oncogenes (OGs) and tumour suppressor genes (TSGs) overlapping with structural variants (SVs) in simulated data with simple breaks that are shown in Fig. 2a.

**type**	**name**	**total copy number**	**#cells (neutral model)**	**#cells (selection model)**
**OG**	*CARD11*	3	3	5
		5	4	6
		7	3	0
		9	0	3
	EGFR	3	7	10
		4	3	3
		5	0	2
	RAC1	3	3	5
		5	4	6
		7	3	0
		9	0	3
**TSG**	*PMS2*	1	86	81
	SFRP4	1	86	87

Supplementary Table 3: The numbers of complex structural variants (SVs), including whole genome doubling (WGD), chromothripsis, extrachromosomal circular DNAs (ecDNAs), and seismic amplifications, detected in simulated data with local fragmentation that are shown in Supplementary Fig. 1 and when increasing probabilities of WGD were introduced in the simulations.

**model**	**#cells with WGD (time appearing)**	**#cells with chromothripsis (time appearing)**	**#cells with ecDNAs (time appearing)**	**#cells with seismic amplifications (time appearing)**	**#cells with chromothripsis, seismic amplification, and ecDNAs**
**neutral**	0	37 ($N = 7$)	95 ($N = 7$)	12 ($N = 43$)	3
**selection**	0	36 ($N = 7$)	95 ($N = 7$)	8 ($N = 43$)	5
**probability of WGD 0.1**					
**neutral**	16 ($N = 16$)	40 ($N = 7$)	98 ($N = 7$)	15 ($N = 19$)	5
**selection**	18 ($N = 16$)	33 ($N = 7$)	99 ($N = 7$)	28 ($N = 19$)	7
**probability of WGD 0.122**					
**neutral**	63 ($N = 6$)	7 ($N = 6$)	98 ($N = 6$)	6 ($N = 12$)	0
**selection**	63 ($N = 6$)	7 ($N = 6$)	98 ($N = 6$)	6 ($N = 12$)	0
**probability of WGD 0.124**					
**neutral**	100 ($N = 2$)	8 ($N = 22$)	100 ($N = 4$)	29 ($N = 13$)	4
**selection**	100 ($N = 2$)	10 ($N = 22$)	100 ($N = 4$)	24 ($N = 13$)	1

Supplementary Table 4: The total copy numbers of oncogenes (OGs) and tumour suppressor genes (TSGs) involved in genome rearrangements in simulated data with local fragmentation that are shown in Supplementary Fig. 1.

type	name	total copy number	#cells (neutral model)	#cells (selection model)
OG	*ACVR1*	3	10	9
		5	10	7
		7	3	3
		9	0	3
		11	3	0
	CTNNA2	3	2	2
		5	6	3
		7	2	0
		11	0	2
		17	2	0
		23	0	2
	CXCR4	3	11	12
		5	6	4
		7	0	2
		13	2	0
	IDH1	3	9	4
		5	2	2
		7	3	0
	MYCN	3	2	2
		5	6	3
		7	2	0
		11	0	2
		17	2	0
		23	0	2
	SF3B1	3	3	6
		5	9	3
		7	3	2
	SIX2	3	2	2
		5	6	3
		7	2	0
		11	0	2
		17	2	0
		23	0	2
	XPO1	3	2	2
		5	6	3
		7	2	0
		11	0	2
		17	2	0
		23	0	2
TSG	*ACVR2A*	1	68	68
	ASXL2	1	85	89
	BARD1	1	78	87
	CASP8	1	80	87
	DNMT3A	1	85	89
	LRP1B	1	73	73
	MSH2	1	85	89
	MSH6	1	85	89
	TMEM127	1	64	76

Supplementary Table 5: The major summary statistics generated by the stochastic cell-cycle model.

**summary statistics**	**range**	**level**	**status in inference**
number of extrachromosomal circular DNAs (ecDNAs)	$[0, \infty]$	per cell	not used
number of chromosome fusions	$[0, \infty]$	per cycle	not used
percentage of genome altered (PGA)	[0,1]	across all cells	used
mean and standard deviation of pairwise divergence	[0,1]	across all cells	used
frequency distribution of breakpoints	[0,1]	across all cells	used
fraction of cells with whole genome doubling (WGD)	[0,1]	across all cells	used
fraction of different types of structural variants (SVs)	[0,1]	across all cells	used

Supplementary Figures

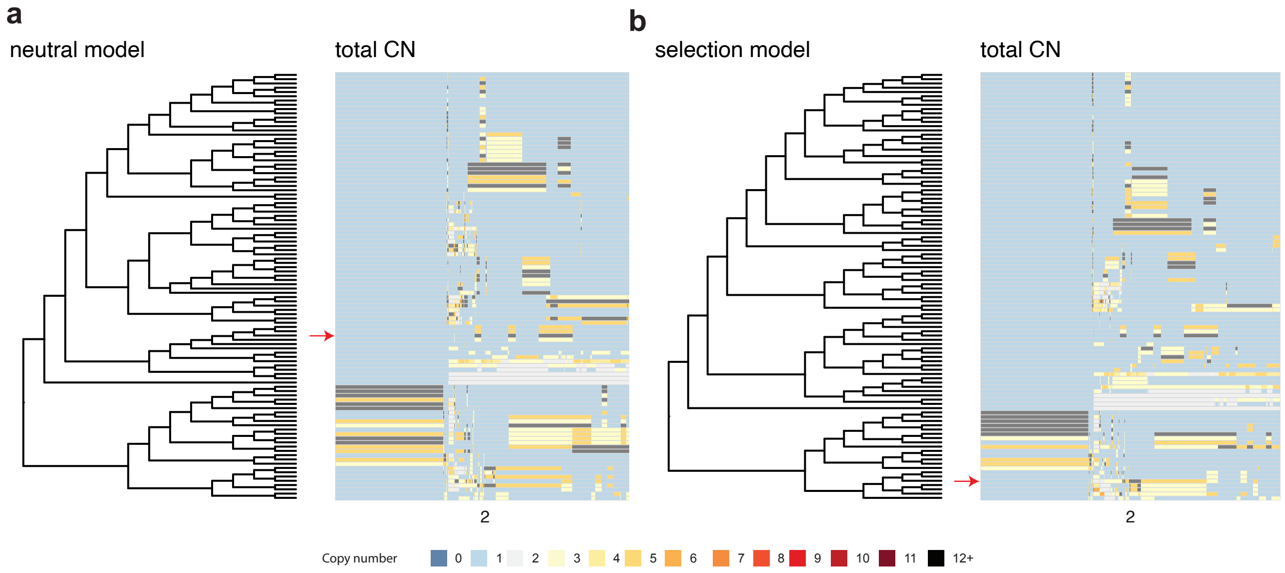

Supplementary Fig. 1: **Cell lineage tree of 100 cells and their corresponding copy numbers on chr2 from the simulation with local fragmentation under neutral evolution (a) and selection (b).** The red arrow in **a** indicates one cell with chromothripsis that is shown in Fig. 2d, generated after eight cell cycles under neutral evolution. The red arrow in **b** indicates one cell with seismic amplification that is shown in Fig. 2e, generated after five cell cycles under selection. Source data are provided as a Source Data file.

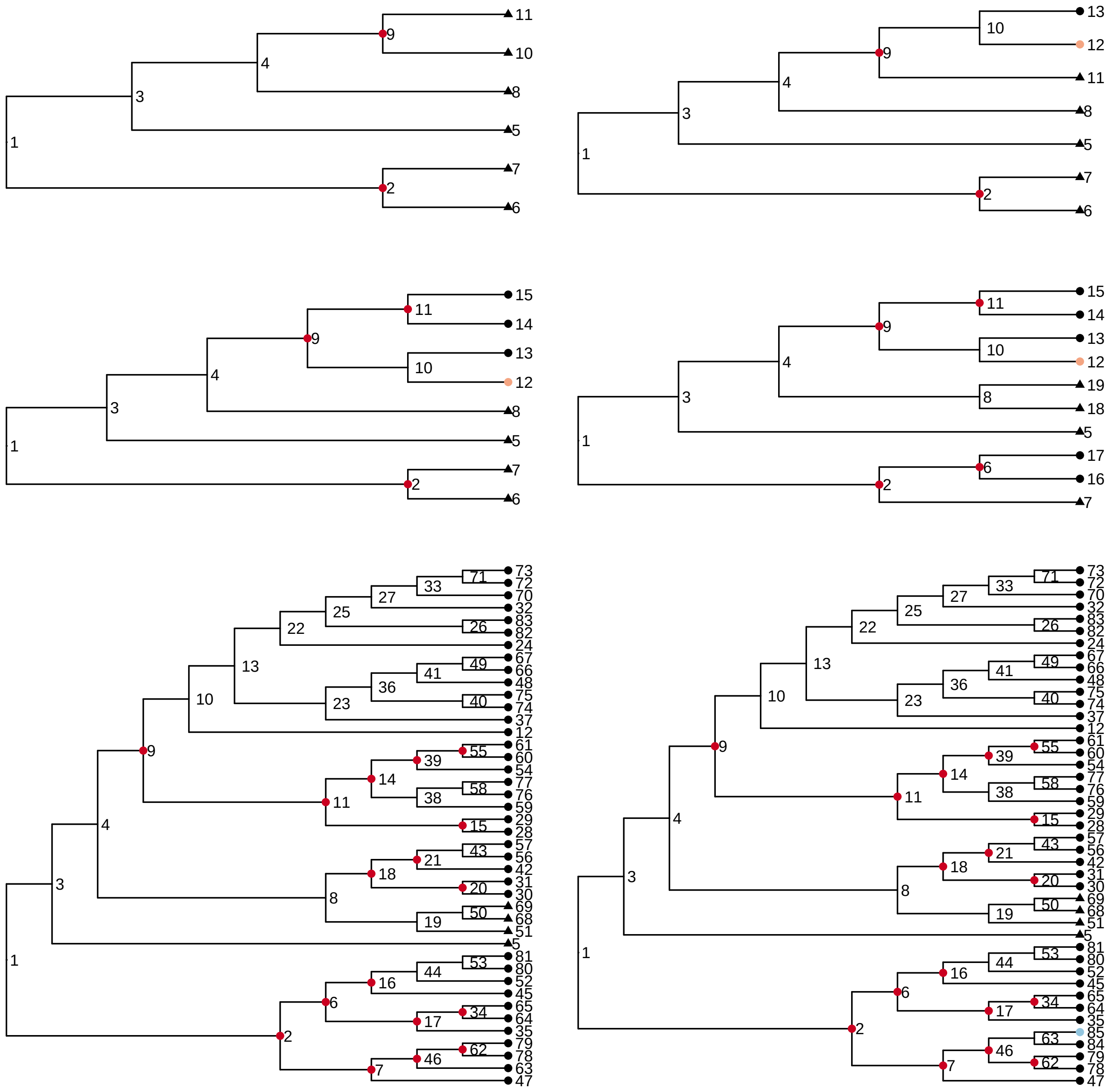

Supplementary Fig. 2: **Cell lineage tree for simulation data in Supplementary Fig. 1 when the population size is 6, 7, 8, 10, 42, and 43, respectively.** The red nodes indicate ancestral cells undergoing local fragmentation. The circular leaves indicate cells with extrachromosomal circular DNAs (ecDNAs). The orange leaves indicate cells with chromothripsis. The blue leaves indicate cells with seismic amplification. The lineages under neutral and selection models are the same. The cell lineage trees are identical under neutral evolution and selection. These cell lineage trees and the observed numbers and timing of complex structural variants (SVs) over time suggest that: 1) chromothripsis and ecDNAs tend to occur concurrently following local fragmentation; 2) all cells exhibiting chromothripsis contain ecDNAs and originate from ancestral cells that underwent local fragmentation; 3) ancestors of cells lacking ecDNAs show little to no local fragmentation. Source data are provided as a Source Data file.

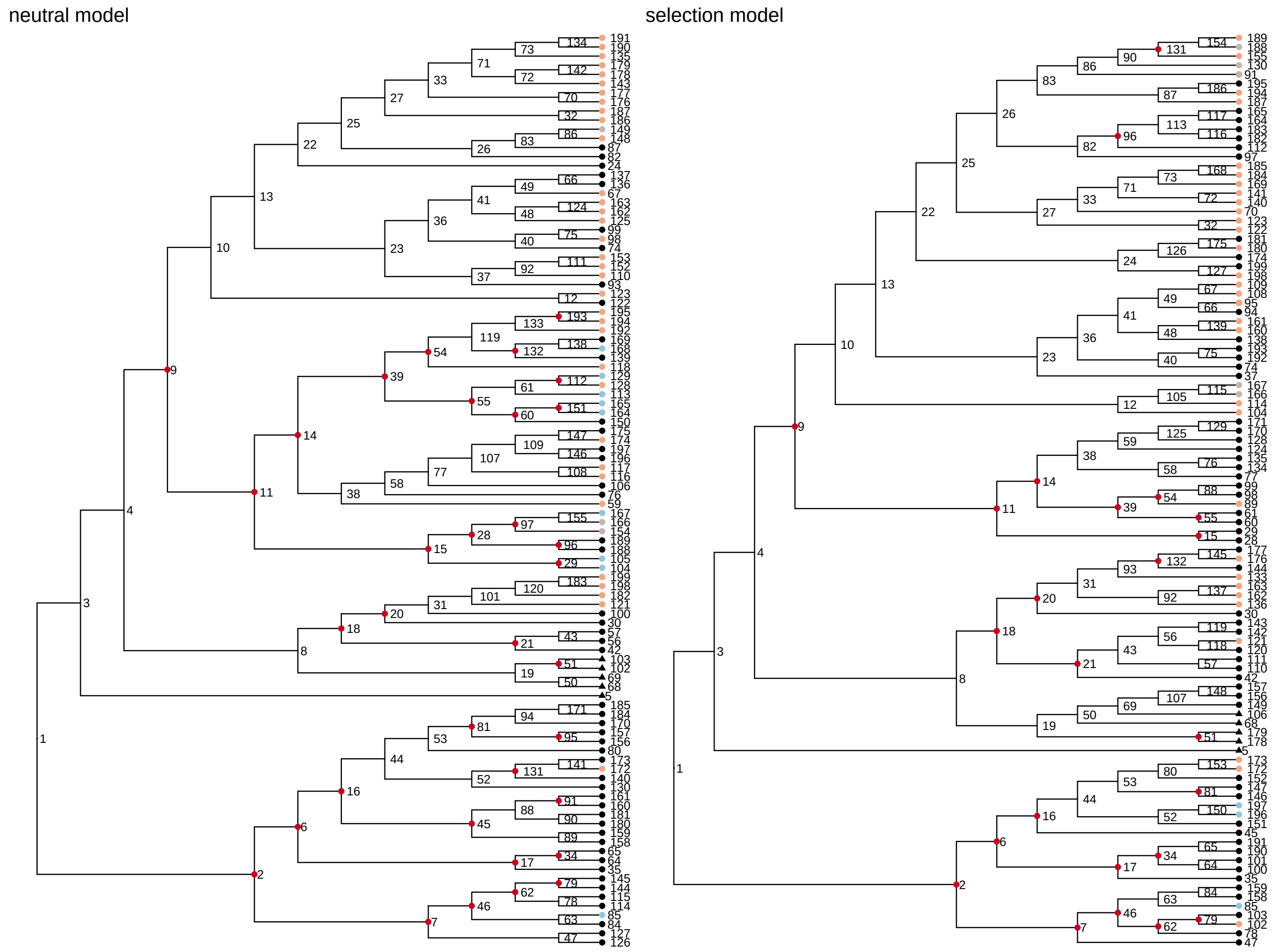

Supplementary Fig. 3: **Cell lineage tree for simulations in Supplementary Fig. 1 under neutral evolution and selection when the population size is 100.** The red nodes indicate ancestral cells undergoing local fragmentation. The circular leaves indicate cells with extrachromosomal circular DNAs (ecDNAs). The orange leaves indicate cells with chromothripsis. The blue leaves indicate cells with seismic amplification. The grey leaves indicate cells with both chromothripsis and seismic amplification. Source data are provided as a Source Data file.

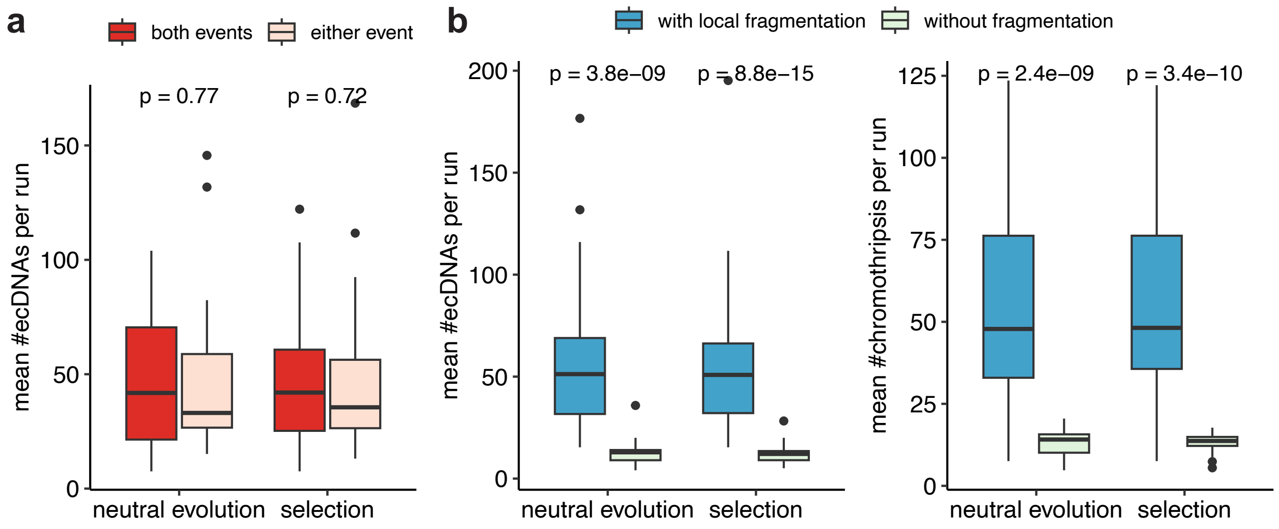

Supplementary Fig. 4: **Mean number of extrachromosomal circular DNAs (ecDNAs) or chromothripsis per run under different conditions. a,** The numbers are grouped according to whether both chromothripsis and ecDNAs were detected in the cell or either event was detected. **b,** The numbers are grouped according to whether local fragmentation were detected in the ancestors of a cell. 50 simulations were generated using the same parameters as in Supplementary Fig. 1 but with different random seeds. Each simulation was run until reaching 100 cells. The p-values are shown for two-sided Wilcoxon test. The box plots show the median (centre), 1st (lower hinge), and 3rd (upper hinge) quartiles of the data; the whiskers extend to 1.5 times of the interquartile range (distance between the 1st and 3rd quartiles); data beyond the interquartile range are plotted individually. Source data are provided as a Source Data file.

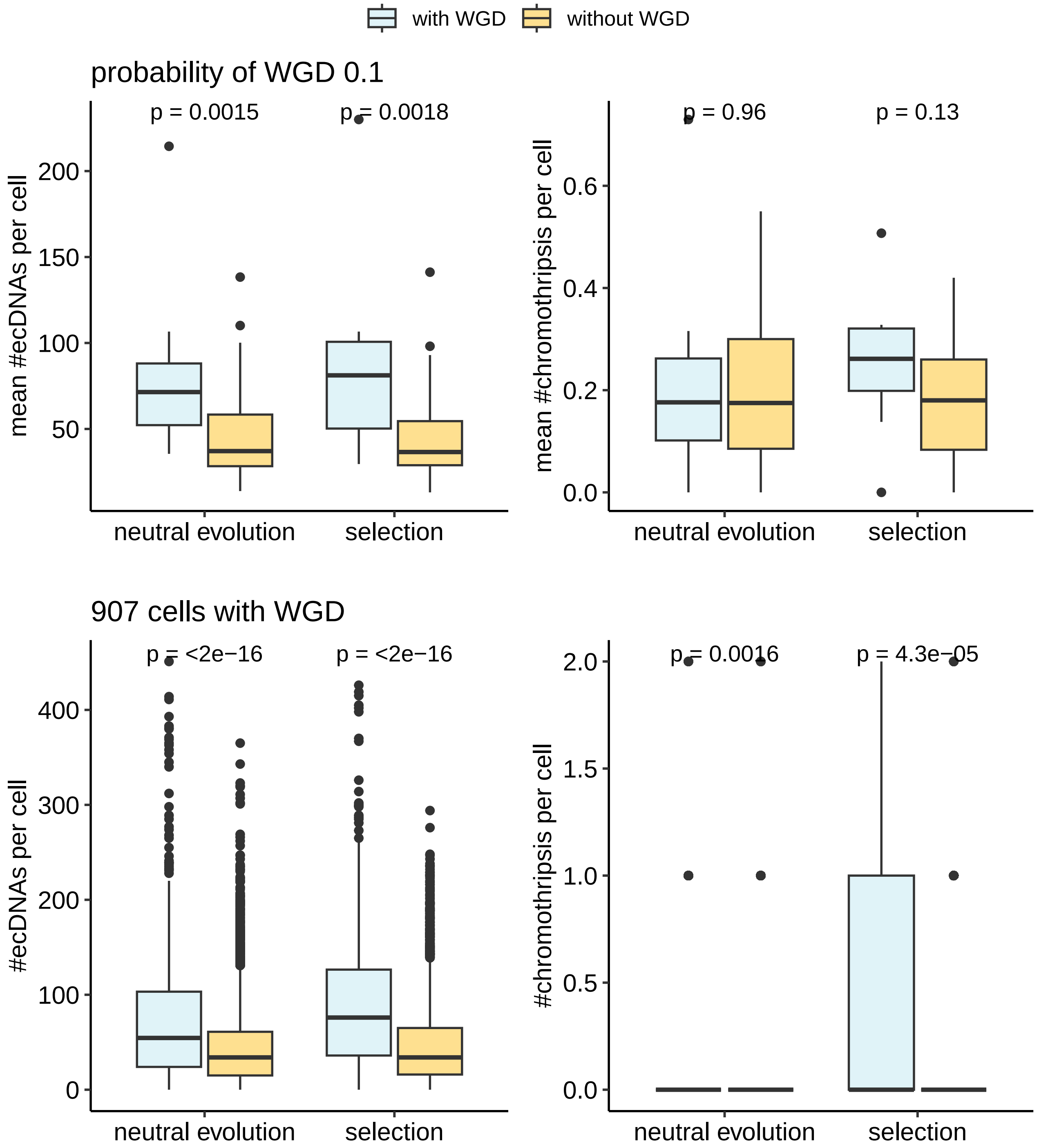

Supplementary Fig. 5: **Mean and absolute number of extrachromosomal circular DNAs (ecDNAs) or chromothripsis per cell when the probability of whole genome doubling (WGD) per cell is 0.1.** 50 simulations were generated using the same parameters as in Supplementary Fig. 1 but with different random seeds. Each simulation was run until reaching 100 cells. There are 456 cells with WGD and 4,544 cells without WGD under neutral evolution. There are 451 cells with WGD and 4,549 cells without WGD under selection. The p-values are shown for two-sided Wilcoxon test. The box plots show the median (centre), 1st (lower hinge), and 3rd (upper hinge) quartiles of the data; the whiskers extend to 1.5 times of the interquartile range (distance between the 1st and 3rd quartiles); data beyond the interquartile range are plotted individually. Source data are provided as a Source Data file.

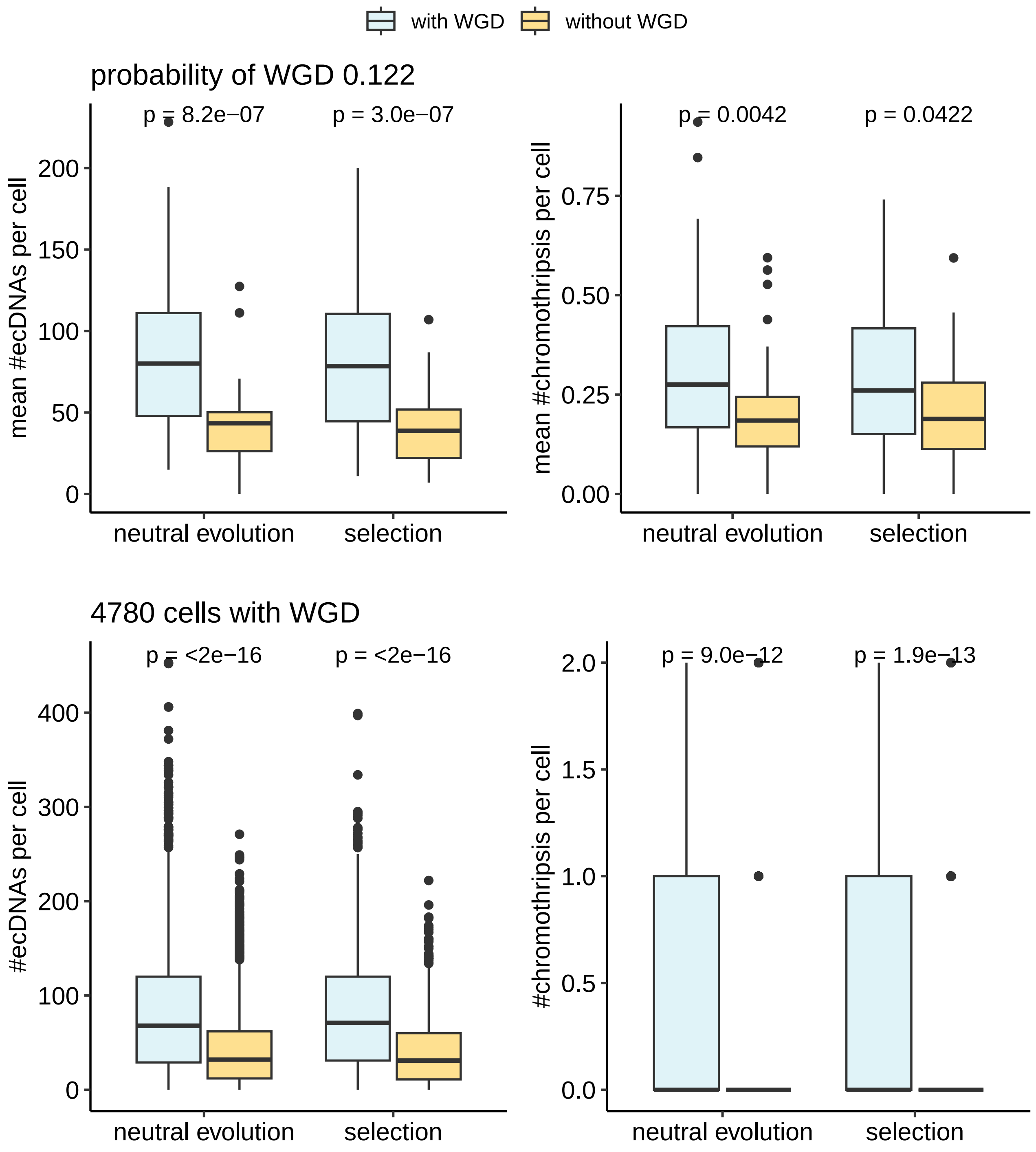

Supplementary Fig. 6: **Mean and absolute number of extrachromosomal circular DNAs (ecDNAs) or chromothripsis per cell when the probability of whole genome doubling (WGD) per cell is 0.122.** 50 simulations were generated using the same parameters as in Supplementary Fig. 1 but with different random seeds. Each simulation was run until reaching 100 cells. There are 2,309 cells with WGD and 2,691 cells without WGD under neutral evolution. There are 2,471 cells with WGD and 2,529 cells without WGD under selection. The p-values are shown for two-sided Wilcoxon test. The box plots show the median (centre), 1st (lower hinge), and 3rd (upper hinge) quartiles of the data; the whiskers extend to 1.5 times of the interquartile range (distance between the 1st and 3rd quartiles); data beyond the interquartile range are plotted individually. Source data are provided as a Source Data file.

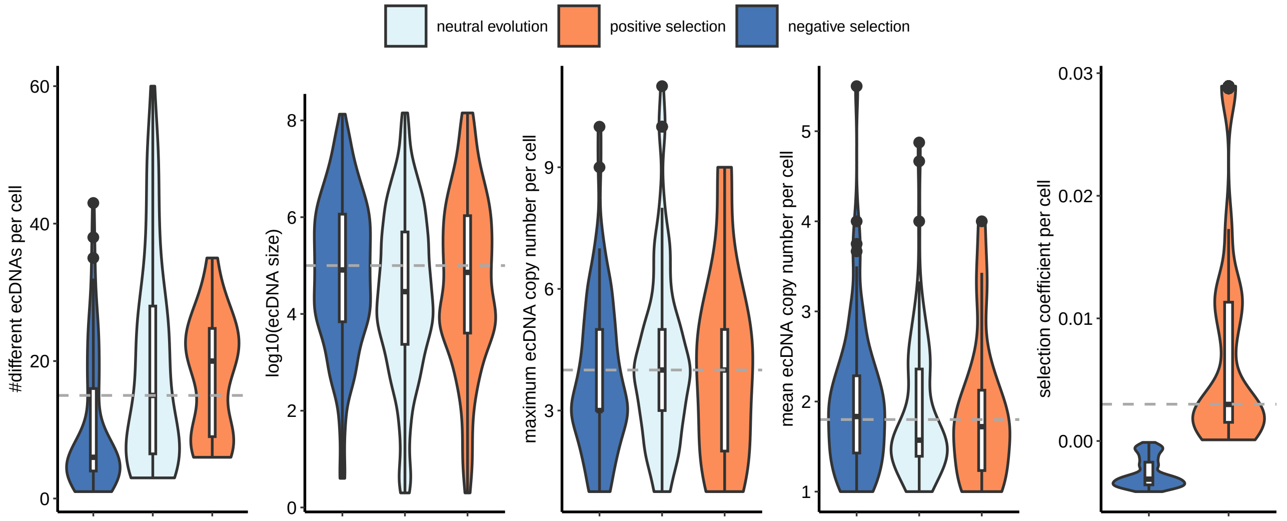

Supplementary Fig. 7: **Distribution of the summary statistics for extrachromosomal circular DNAs (ecDNAs) from the simulation data with local fragmentation that are shown in Supplementary Fig. 1.** 73, 95, and 22 cells contain 753, 1,857, and 399 ecDNAs under negative selection, neutral evolution, and positive selection, respectively. The dashed lines are used as references for comparison. The box plots show the median (centre), 1st (lower hinge), and 3rd (upper hinge) quartiles of the data; the whiskers extend to 1.5 times of the interquartile range (distance between the 1st and 3rd quartiles); data beyond the interquartile range are plotted individually. Source data are provided as a Source Data file.

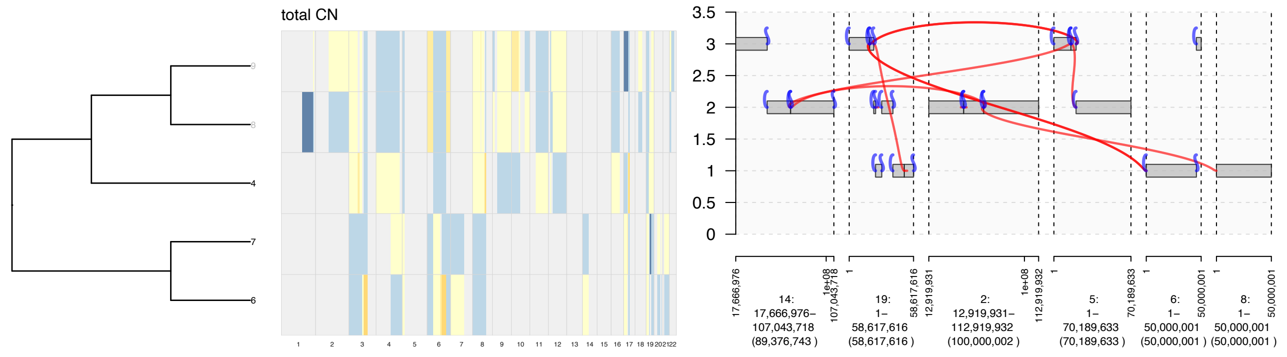

Supplementary Fig. 8: **Cell lineage tree of five cells and their corresponding copy number heatmap across all chromosomes from the simulation with multiple misrepaired double-strand breaks (DSBs), as well as a detected chromoplexy in cell 6.** The cells without chromoplexy are shown in grey. Source data are provided as a Source Data file.

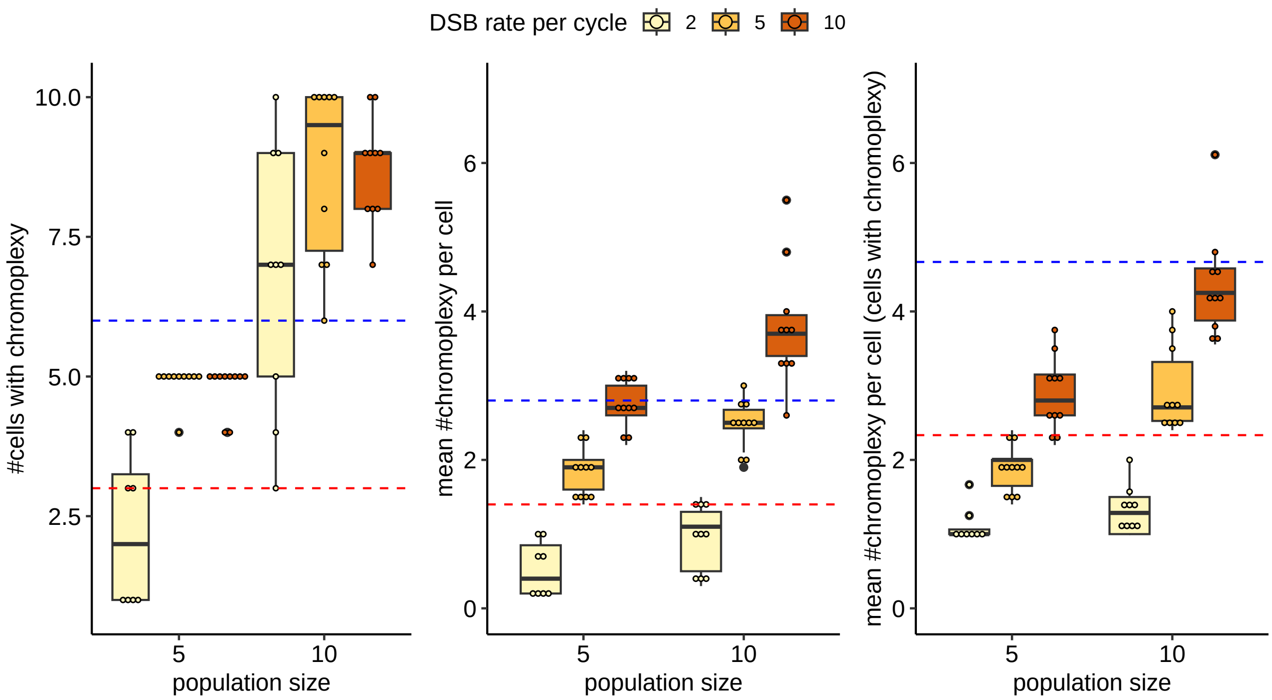

Supplementary Fig. 9: **Summary statistics of cells with chromoplexy under different double-strand break (DSB) rates per cycle.** The red dashed line indicates the observed value in Supplementary Fig. 8 when there are 5 cells. The blue dashed line indicates the observed value in Fig. 2f when there are 10 cells. There are 10 data points for each parameter setting. The box plots show the median (centre), 1st (lower hinge), and 3rd (upper hinge) quartiles of the data; the whiskers extend to 1.5 times of the interquartile range (distance between the 1st and 3rd quartiles); data beyond the interquartile range are plotted individually. Source data are provided as a Source Data file.

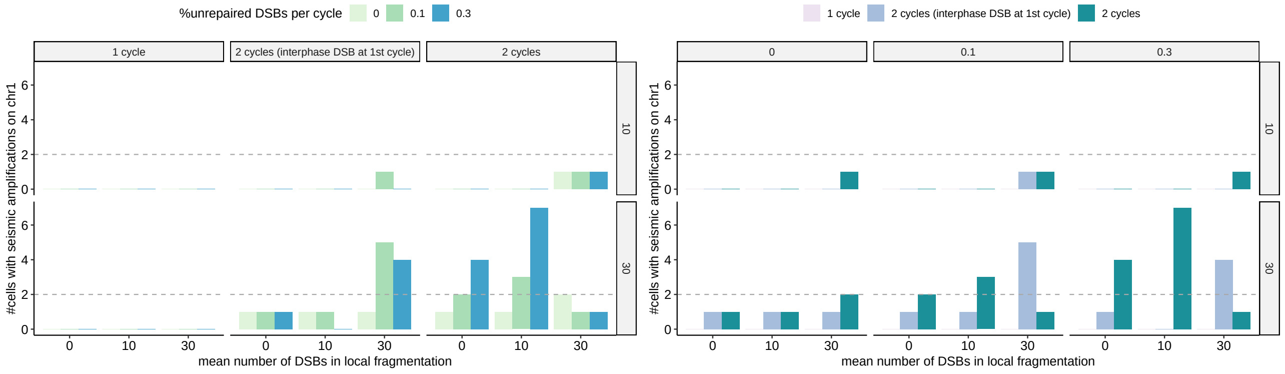

Supplementary Fig. 10: **Number of cells with seismic amplifications on chr1 under different parameter settings in one or two cell cycles.** The labels at the right of each plot indicate double-strand break (DSB) rates per cycle. The plots correspond to the same simulation data presented in Fig. 3b-e. Source data are provided as a Source Data file.

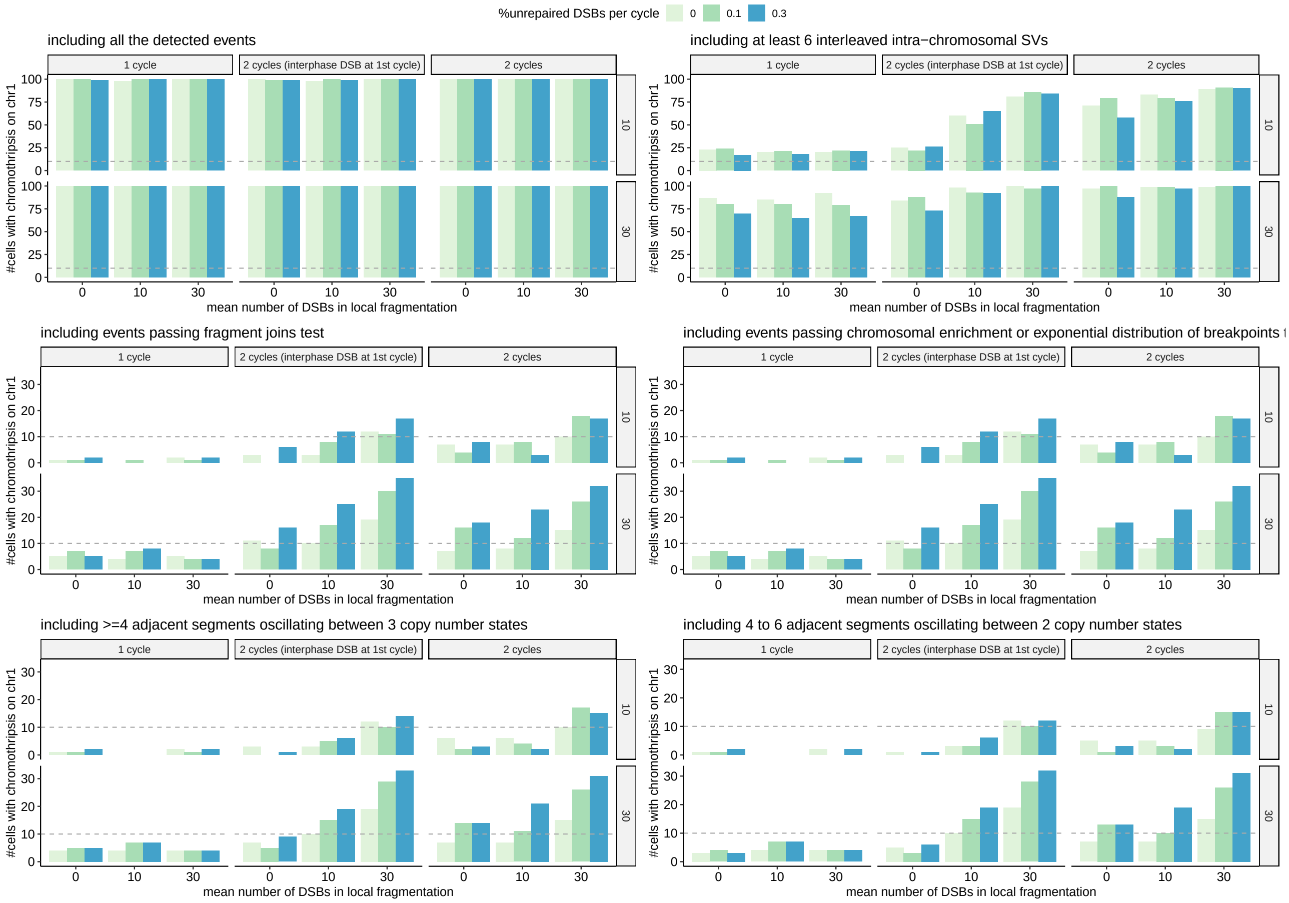

Supplementary Fig. 11: **Number of detected chromothripsis events decreases with the application of successive criteria.** The criteria employed in detecting high-confidence chromothripsis events by ShatterSeek include: 1) at least seven adjacent segments oscillating between two copy number states, 2) at least six interleaved intra-chromosomal structural variants (SVs), 3) fragment joins test, and 4) either the chromosomal enrichment test or the exponential distribution of breakpoints test. The results of including two less stringent criteria on the number of adjacent segments oscillating between copy number states are shown in the bottom two panels. The labels at the right of each plot indicate double-strand break (DSB) rates per cycle. The plots correspond to the same simulation data presented in Fig. 3b-e. Source data are provided as a Source Data file.

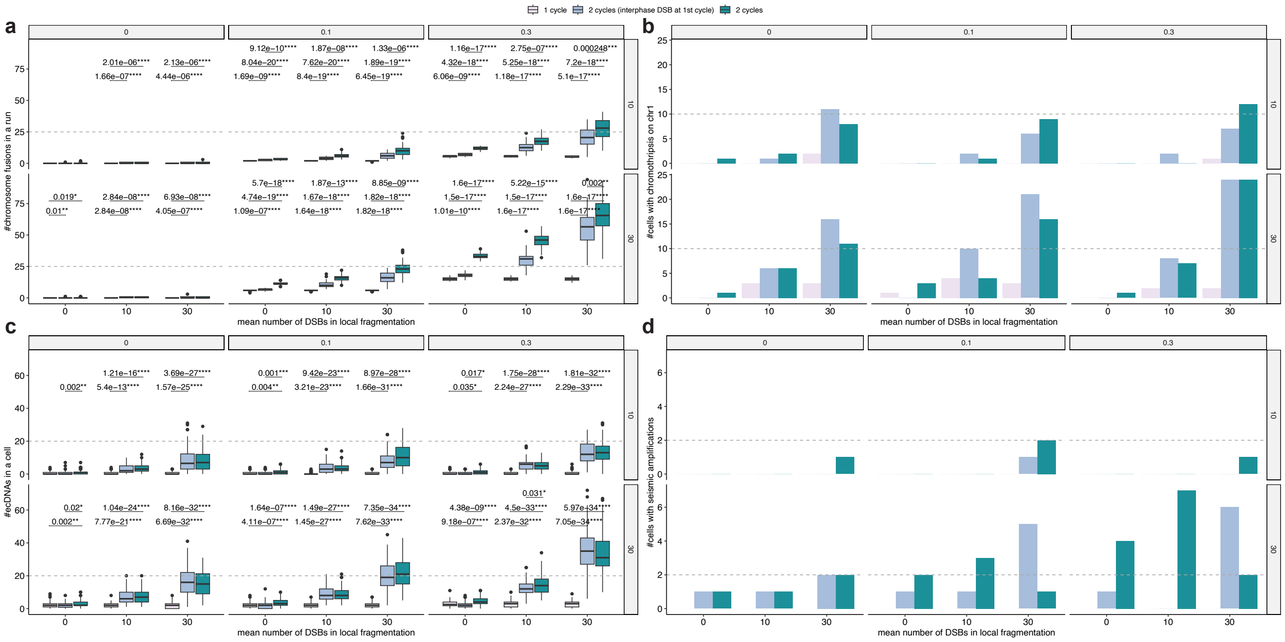

Supplementary Fig. 12: **Distribution of complex structural variants (SVs) under different parameter settings in one or two cell cycles shown in groups different from those in Fig. 3. a,** The number of chromosome fusions in a run of simulation under different parameter settings in one or two cell cycles. Each box plot contains 50 data points, corresponding to 50 simulations under the same parameter setting. **b,** The number of cells with chromothripsis on chr1 under different parameter settings in one or two cell cycles. **c,** The number of extrachromosomal circular DNAs (ecDNAs) in a cell under different parameter settings in one or two cell cycles. Each box plot contains 100 data points, corresponding to 100 cells across 50 simulations under the same parameter setting. **d,** The number of cells with seismic amplifications under different parameter settings in one or two cell cycles. The labels at the top of each plot indicate fractions of unrepaired double-strand breaks (DSBs) per cycle, whereas the labels at the right indicate DSB rates per cycle. For each run, we consider the two cells at the end of either the first or second cycle depending on the number of cycles. The box plots show the median (centre), 1st (lower hinge), and 3rd (upper hinge) quartiles of the data; the whiskers extend to 1.5 times of the interquartile range (distance between the 1st and 3rd quartiles); data beyond the interquartile range are plotted individually. The significance levels of significant p-values from two-sided Wilcoxon tests are shown: * – p-val < 0.05, ** – p-val < 0.01, *** – p-val < 0.001, **** – p-val < 0.0001. The p-values were adjusted by Bonferroni correction for multiple pairwise tests. The two endpoints of the horizontal line below the p-value represent the two groups being compared. Source data are provided as a Source Data file.

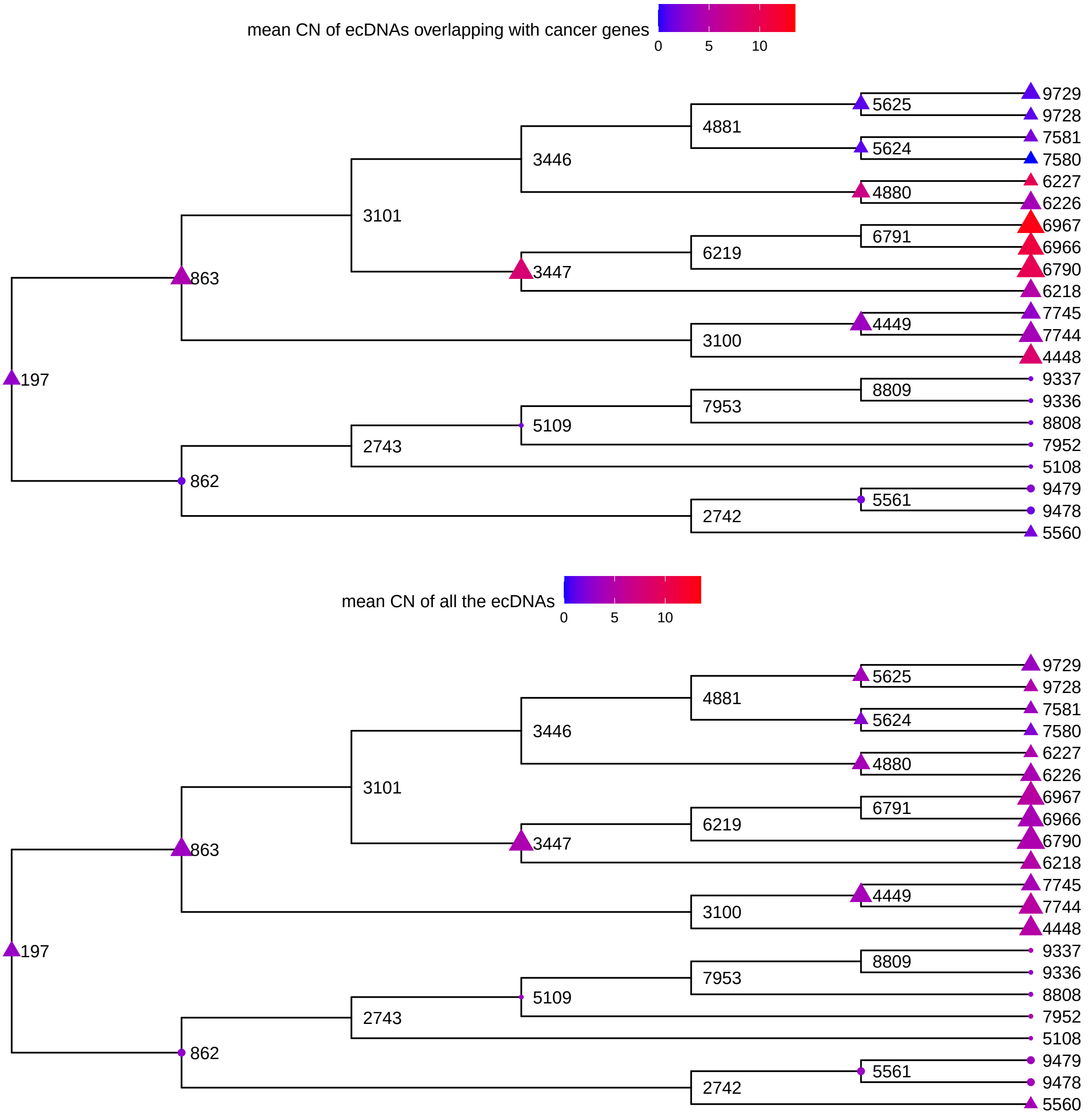

Supplementary Fig. 13: **Lineage of cell 197 with one extrachromosomal circular DNA (ecDNA) carrying cancer genes under selection indicates fitness changes of ecDNAs with different copy numbers.** Cell 197 was extracted from the available cells when the total number of cells reached 100 as in the simulated lineages presented in Supplementary Fig. 1b. Cell 197 gave rise to two daughter cells (ID: 862 and 863), Node size indicates the probability of survival, whereas node shape indicates the type of selection (round: negative, triangle: positive). Cell 862 had one ecDNA with a single copy and the other with two copies. Cell 863 had one ecDNA with four copies and the other with five copies. When the total number of cells reached 5000, cell 862 had eight descendants, all of which but one underwent negative selection. In contrast, cell 863 had 13 descendants, all of which underwent positive selection. As indicated by node sizes, cells with higher ecDNA copy numbers had a higher probability of survival, but they also sometimes underwent weaker positive selection (e.g., cell 6227 with copy number 10 for ecDNAs overlapping with cancer genes) or negative selection (e.g., cell 9479 with copy number 4 for ecDNAs overlapping with cancer genes). Source data are provided as a Source Data file.

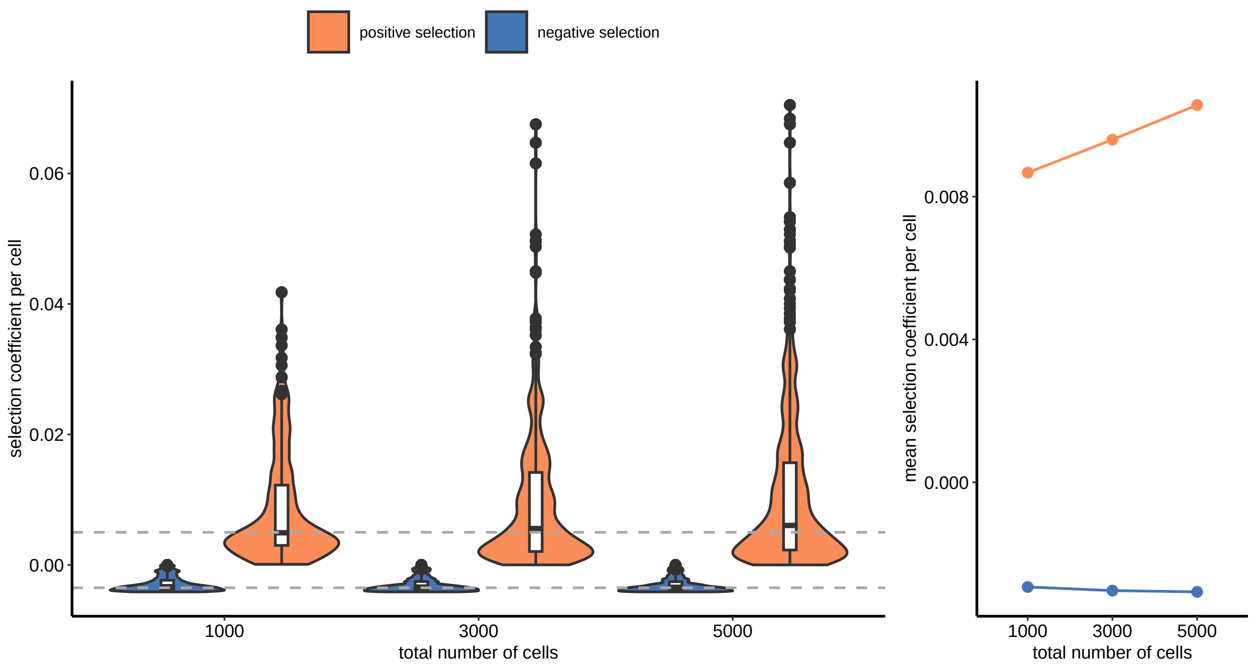

Supplementary Fig. 14: **Distribution of selection coefficients of cells under selection over time.** When the total number of cells is 1,000, 813 and 166 cells are under negative and positive selection, respectively. When the total number of cells is 3,000, 2,416 and 483 cells are under negative and positive selection, respectively. When the total number of cells is 5,000, 3,971 and 809 cells are under negative and positive selection, respectively. The box plots show the median (centre), 1st (lower hinge), and 3rd (upper hinge) quartiles of the data; the whiskers extend to 1.5 times of the interquartile range (distance between the 1st and 3rd quartiles); data beyond the interquartile range are plotted individually. The plots correspond to the same simulation data presented in Fig. 4b-d. Source data are provided as a Source Data file.

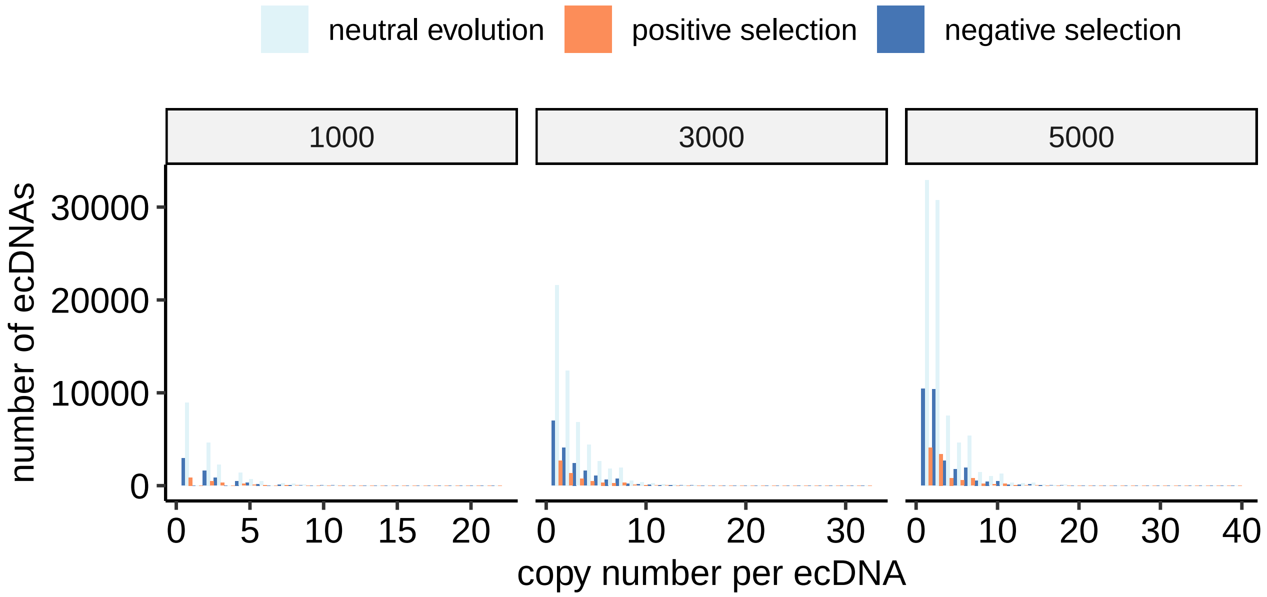

Supplementary Fig. 15: **Number of extrachromosomal circular DNAs (ecDNAs) with different copy numbers across over time.** The label at the top of each plot indicates the total number of cells in the population. The plots correspond to the same simulation data presented in Fig. 4b-d. When the total number of cells is 1,000, 813, 993, and 166 cells contain 6,760, 19,244, and 2,200 ecDNAs under negative selection, neutral evolution, and positive selection, respectively. When the total number of cells is 3,000, 2,416, 2,972, and 483 cells contain 18,411, 53,450, and 6,468 ecDNAs under negative selection, neutral evolution, and positive selection, respectively. When the total number of cells is 5,000, 3,971, 4,939, and 809 cells contain 29,245, 86,315, and 10,477 ecDNAs under negative selection, neutral evolution, and positive selection, respectively. Source data are provided as a Source Data file.

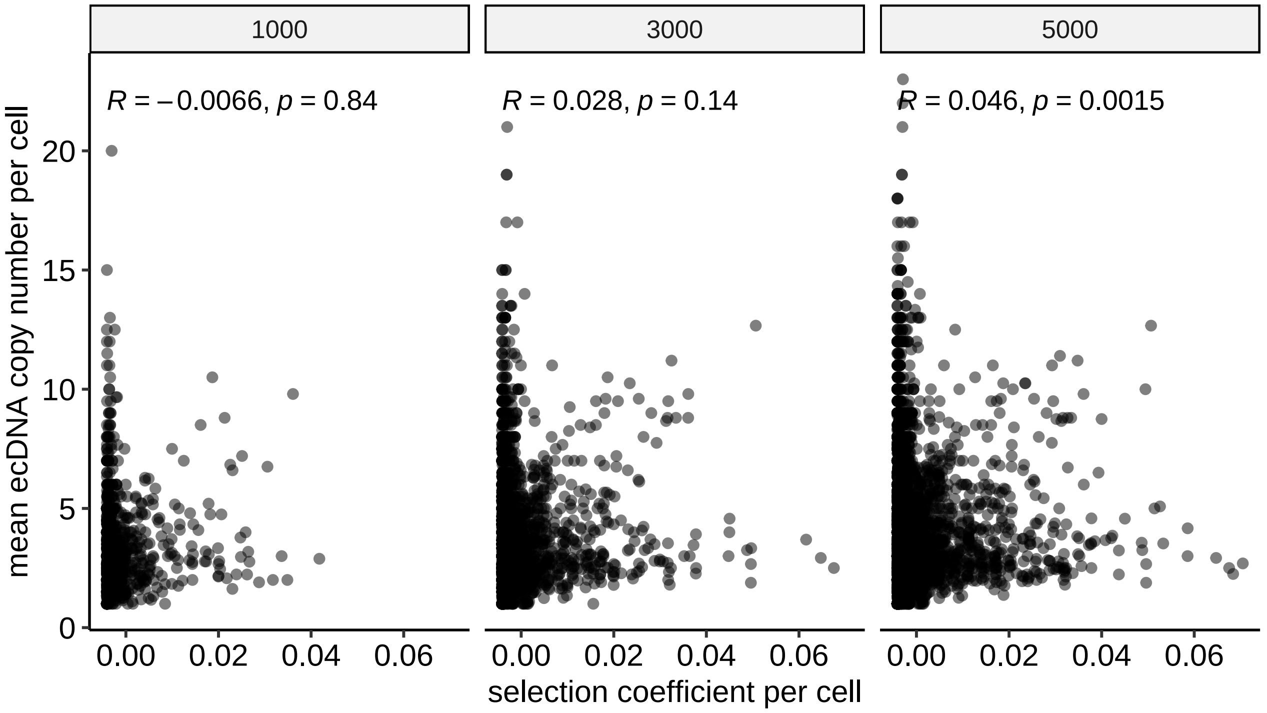

Supplementary Fig. 16: **Correlation between the selection coefficient and mean extrachromosomal circular DNA (ecDNA) copy number of a cell under selection over time.** The Spearman correlation coefficient and corresponding two-sided p-value are shown for each plot. The label at the top of each plot indicates the total number of cells in the population. The plots correspond to the same simulation data presented in Fig. 4b-d. When the total number of cells is 1,000, 813 and 166 cells contain 6,760 and 2,200 ecDNAs under negative and positive selection, respectively. When the total number of cells is 3,000, 2,416 and 483 cells contain 18,411 and 6,468 ecDNAs under negative and positive selection, respectively. When the total number of cells is 5,000, 3,971 and 809 cells contain 29,245 and 10,477 ecDNAs under negative and positive selection, respectively. Source data are provided as a Source Data file.

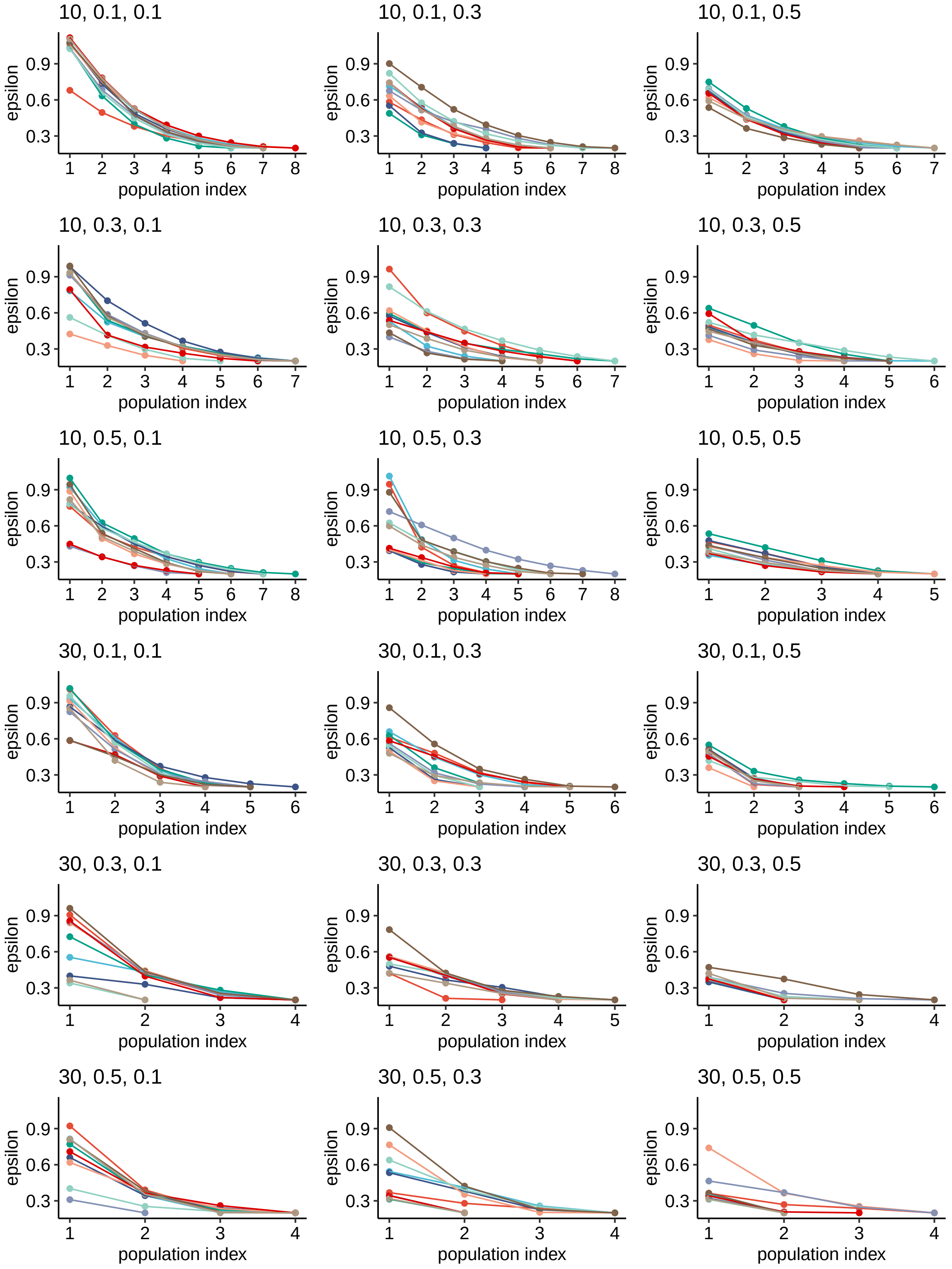

Supplementary Fig. 17: **Tolerance change across populations of approximate Bayesian computation sequential Monte Carlo (ABC SMC) applied to 180 datasets simulated under 18 different parameter settings.** Each panel represents the results for one set of parameters (r, f_u, p_w), where r is double-strand break (DSB) rate per cycle, f_u is fraction of unrepaired DSBs per cycle, and p_w is probability of whole genome doubling (WGD) per cell. Different colors in each plot represent the 10 simulations under the same parameter setting. Source data are provided as a Source Data file.

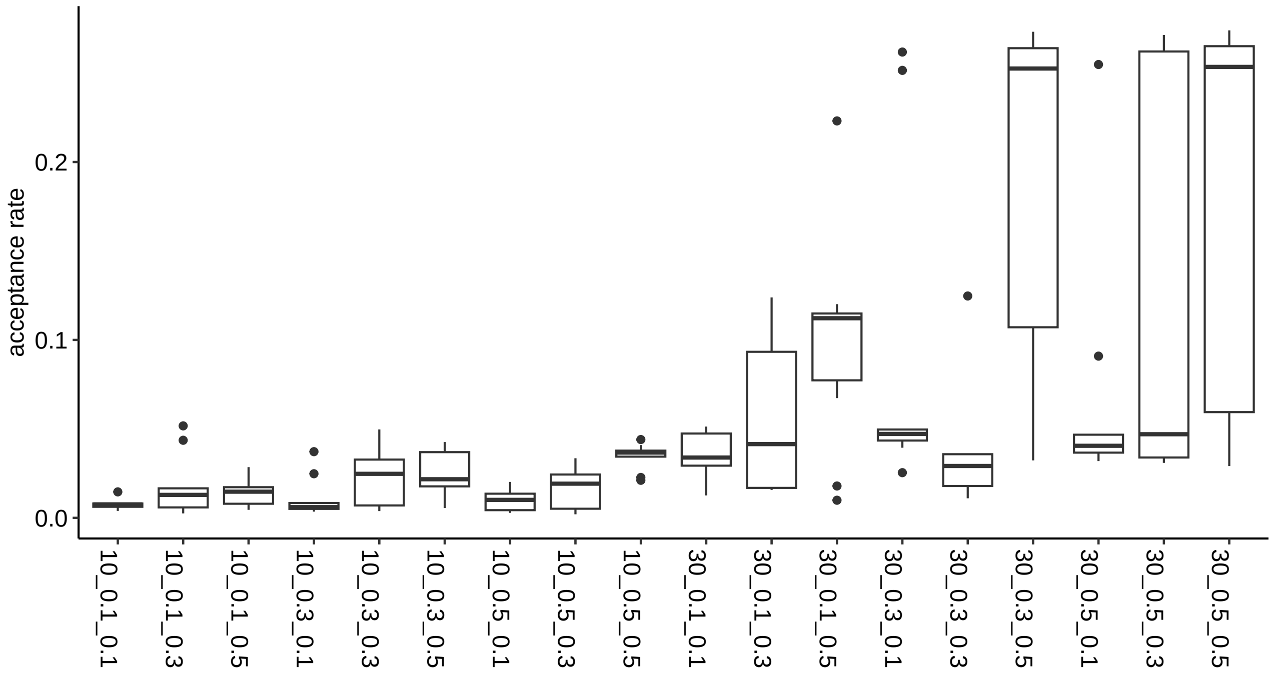

Supplementary Fig. 18: **Acceptance rate of approximate Bayesian computation sequential Monte Carlo (ABC SMC) applied to 180 datasets simulated under 18 different parameter settings.** Each sample is represented by the parameters used for simulation $r\_f_u\_p_w$, where r is double-strand break (DSB) rate per cycle, f_u is fraction of unrepaired DSBs per cycle, and p_w is probability of whole genome doubling (WGD) per cell. The box plots show the median (centre), 1st (lower hinge), and 3rd (upper hinge) quartiles of the data; the whiskers extend to 1.5 times of the interquartile range (distance between the 1st and 3rd quartiles); data beyond the interquartile range are plotted individually. Each box plot contains 10 data points, representing 10 simulations under the same parameter setting. Source data are provided as a Source Data file.

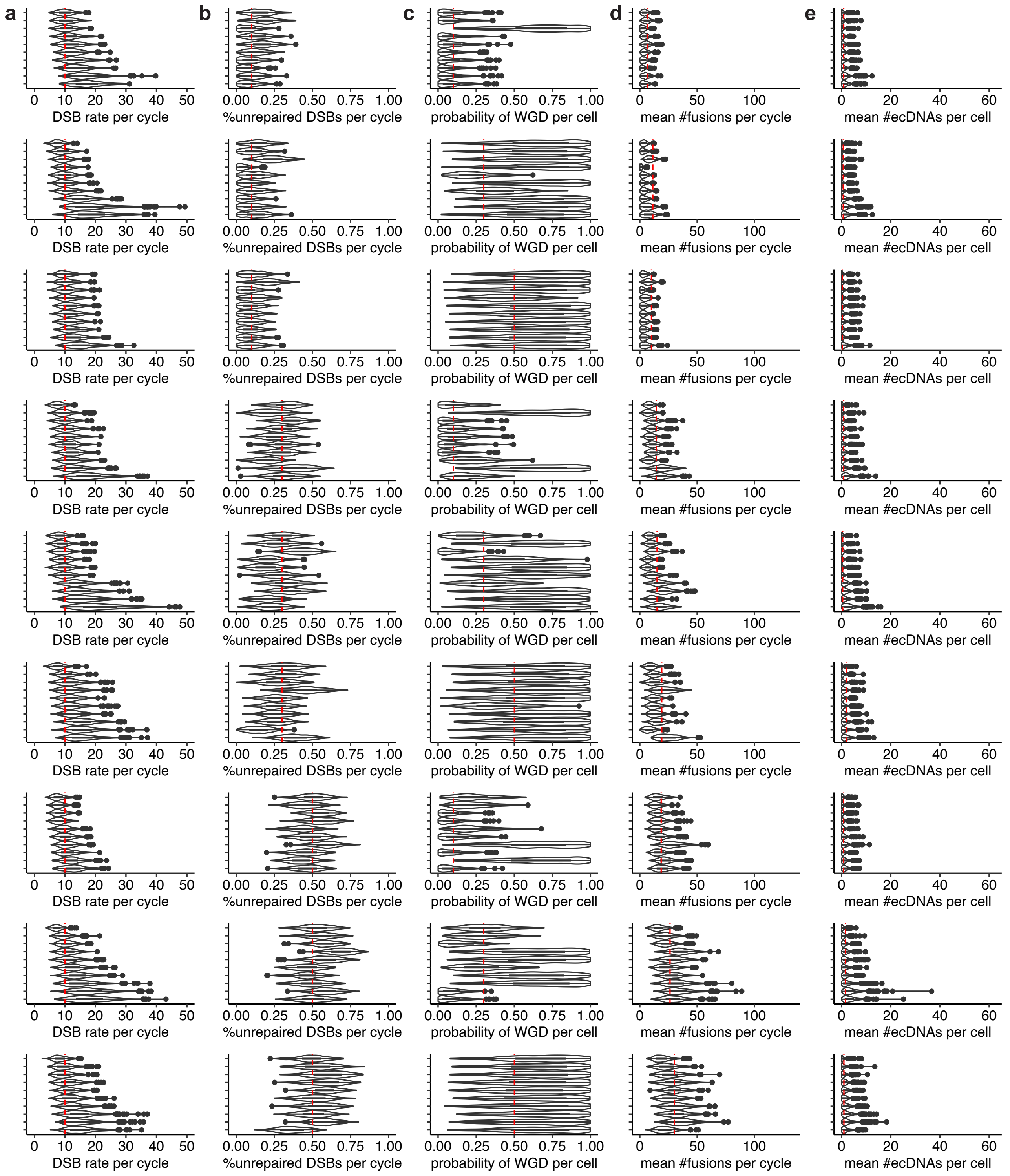

Supplementary Fig. 19: **Posterior distributions of three inferred parameters and the posterior predictive distributions of two summary statistics based on simulated data with double-strand break (DSB) rate per cycle set to $r = 10$.** Each row represents the results for one set of parameters (r, f_u, p_w), where f_u is fraction of unrepaired DSBs per cycle and p_w is probability of whole genome doubling (WGD) per cell. The red dashed line indicates the true parameter value. The results are sorted by the inferred values of r. The violin plots for the three inferred parameters in **a** to **c** are weighted. Each box plot contains 500 data points, which represent the posterior samples. Source data are provided as a Source Data file.

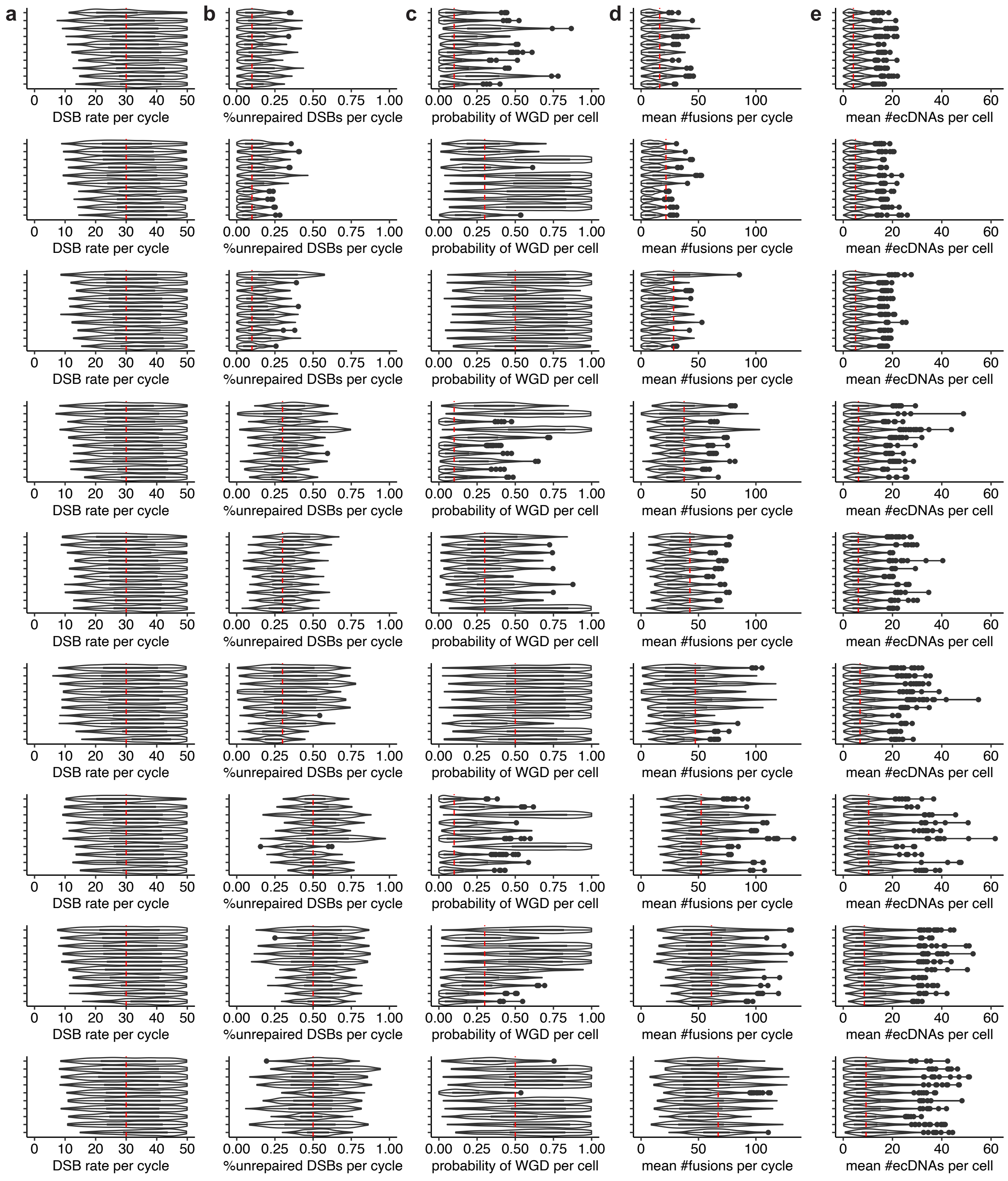

Supplementary Fig. 20: **Posterior distributions of three inferred parameters and the posterior predictive distributions of two summary statistics based on simulated data with double-strand break (DSB) rate per cycle set to** $r = 30$. Each row represents the results for one set of parameters (r, f_u, p_w), where f_u is fraction of unrepaired DSBs per cycle and p_w is probability of whole genome doubling (WGD) per cell. The red dashed line indicates the true parameter value. The results are sorted by the inferred values of r. The violin plots for the three inferred parameters in **a** to **c** are weighted. Each box plot contains 500 data points, which represent the posterior samples. Source data are provided as a Source Data file.

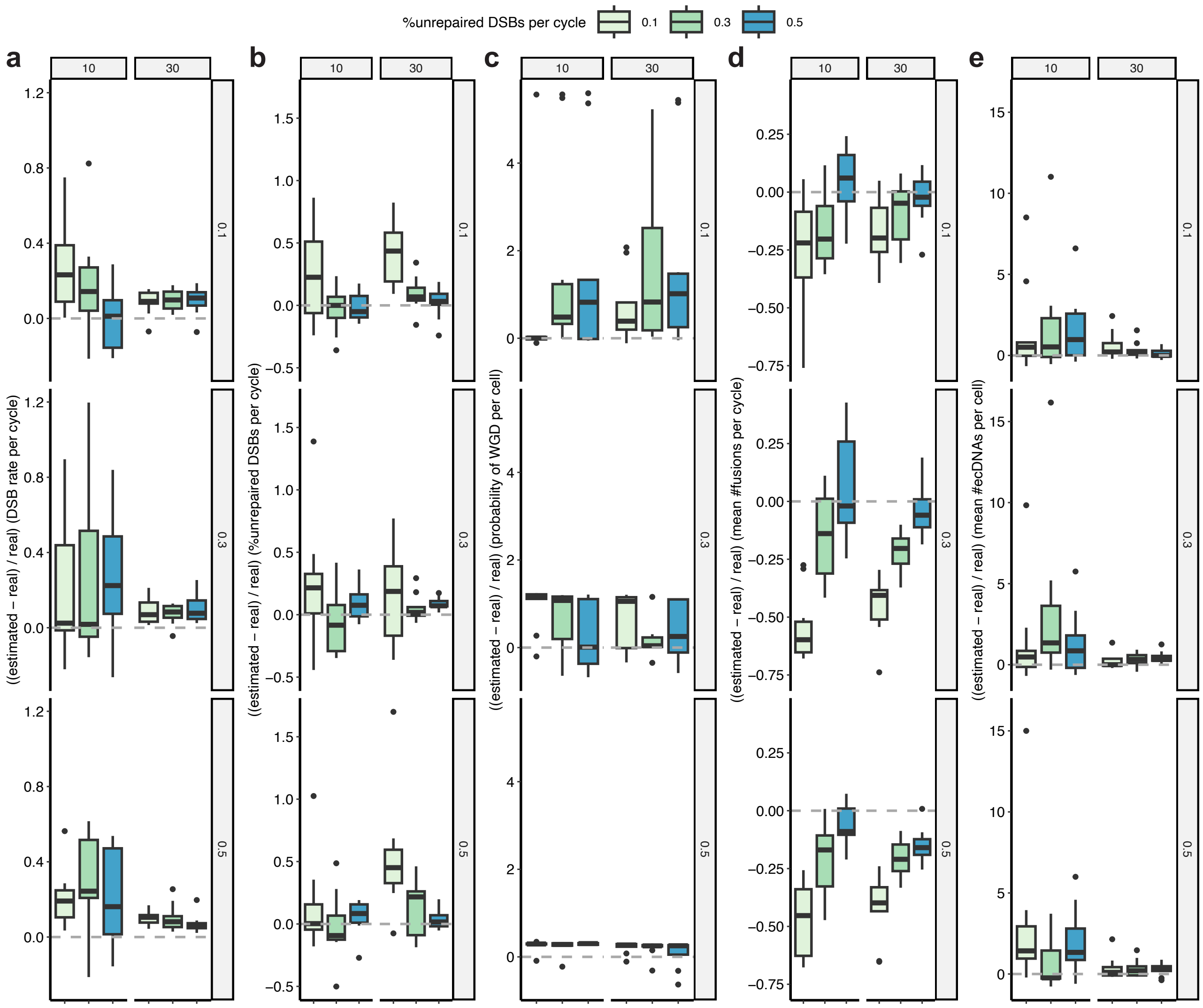

Supplementary Fig. 21: **Distributions of posterior means of parameters inferred using approximate Bayesian computation sequential Monte Carlo (ABC SMC) on simulated data.** **a,** The proportion of differences between estimated and real double-strand break (DSB) rate per cycle. **b,** The proportion of differences between estimated and real fraction of unrepaired DSBs per cycle. **c,** The proportion of differences between estimated and real probability of whole genome doubling (WGD) per cell. **d,** The proportion of differences between estimated and real mean number of chromosome fusions per cycle. **e,** The proportion of differences between estimated and real number of extrachromosomal circular DNAs (ecDNAs) per cell. The labels at the top of each plot show DSB rates per cycle, whereas the labels at the right show probabilities of WGD per cell. The box plots show the median (centre), 1st (lower hinge), and 3rd (upper hinge) quartiles of the data; the whiskers extend to 1.5 times of the interquartile range (distance between the 1st and 3rd quartiles); data beyond the interquartile range are plotted individually. There are 10 data points for each box plot, which represent the posterior means of 10 runs under the same parameter setting. Source data are provided as a Source Data file.

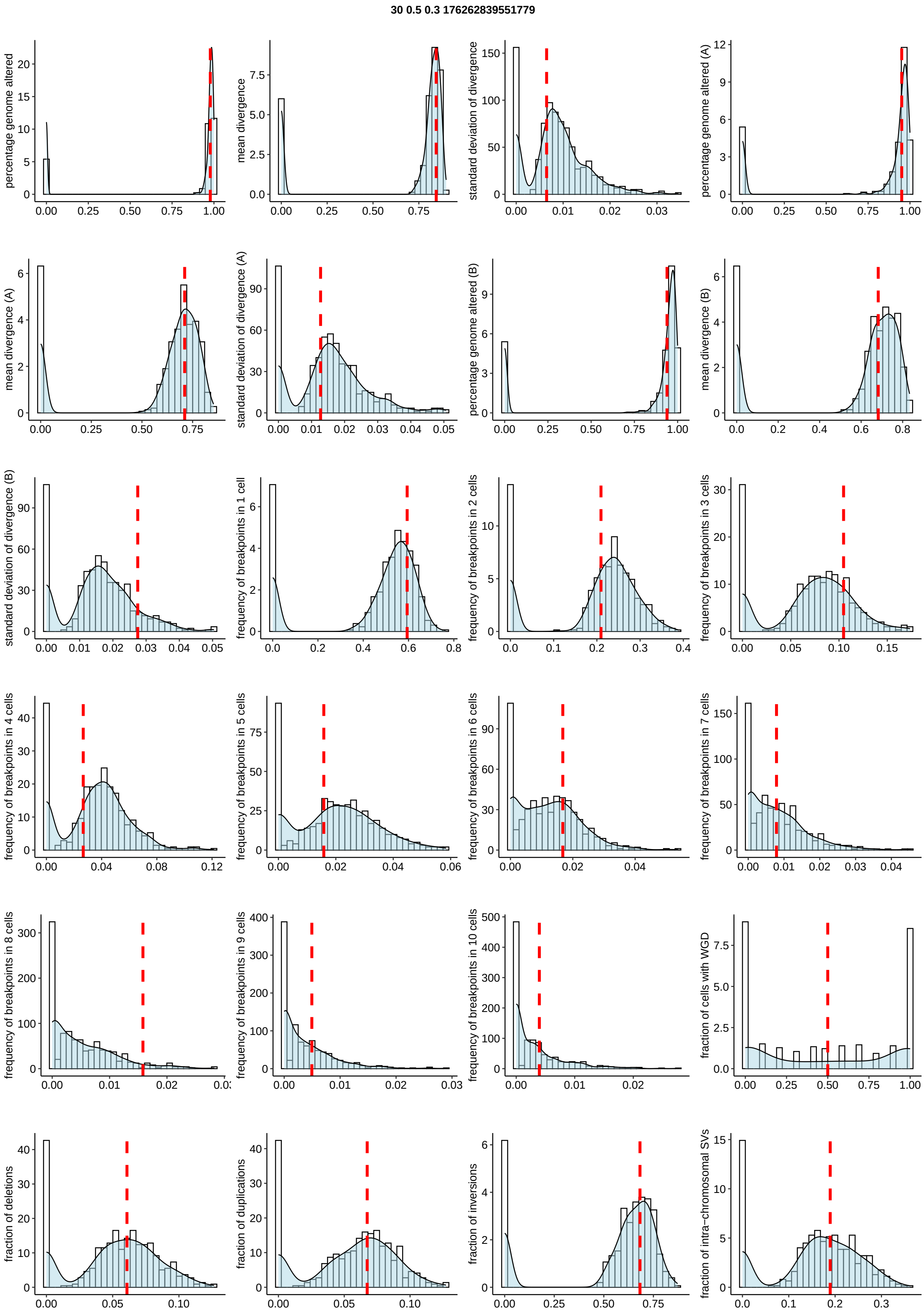

Supplementary Fig. 22: **Posterior predictive distributions of the summary statistics used for inference from a simulated dataset.** The red dashed line indicates the observed value. The title indicates the true parameter values used for simulating the data, where double-strand break (DSB) rate per cycle is $r = 30$, fraction of unrepaired DSBs per cycle is $f_u = 0.5$, probability of whole genome doubling (WGD) per cell is $p_w = 0.3$, and the random seed is 176262839551779. All the observed values fell within two standard deviations of the distributions. Each plot contains 500 data points, which represent the posterior samples. Source data are provided as a Source Data file.

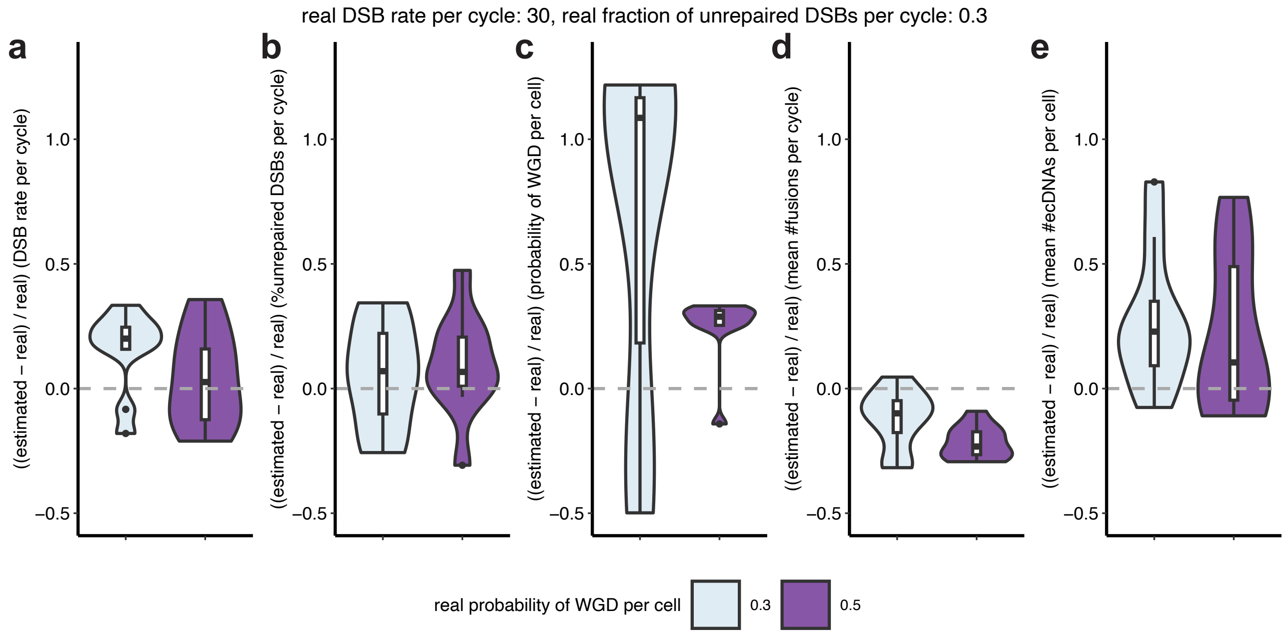

Supplementary Fig. 23: **Accuracy of parameter inference using approximate Bayesian computation sequential Monte Carlo (ABC SMC) on data simulated using breakpoints sampled from a primary breast cancer patient.** **a,** The proportion of differences between estimated and real double-strand break (DSB) rate per cycle. **b,** The proportion of differences between estimated and real fraction of unrepaired DSBs per cycle. **c,** The proportion of differences between estimated and real probability of whole genome doubling (WGD) per cell. **d,** The proportion of differences between estimated and real mean number of chromosome fusions per cycle. **e,** The proportion of differences between estimated and real number of extrachromosomal circular DNAs (ecDNAs) per cell. The box plots show the median (centre), 1st (lower hinge), and 3rd (upper hinge) quartiles of the data; the whiskers extend to 1.5 times of the interquartile range (distance between the 1st and 3rd quartiles); data beyond the interquartile range are plotted individually. There are 10 data points for each box plot, which represent the posterior means of 10 runs under the same parameter setting. Source data are provided as a Source Data file.

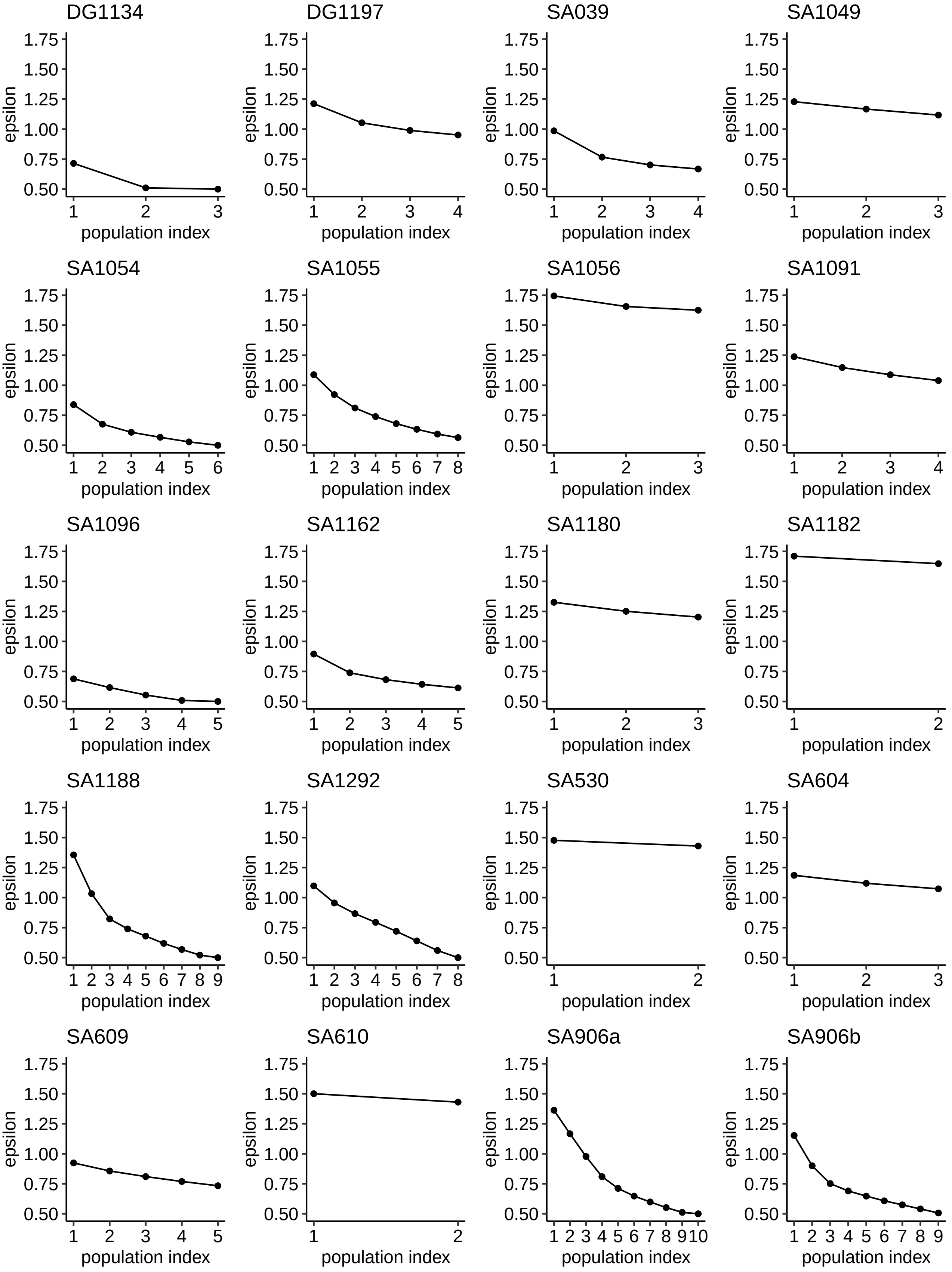

Supplementary Fig. 24: **Tolerance change across populations of approximate Bayesian computation sequential Monte Carlo (ABC SMC) applied to 20 single-cell whole-genome sequencing datasets.** Source data are provided as a Source Data file.

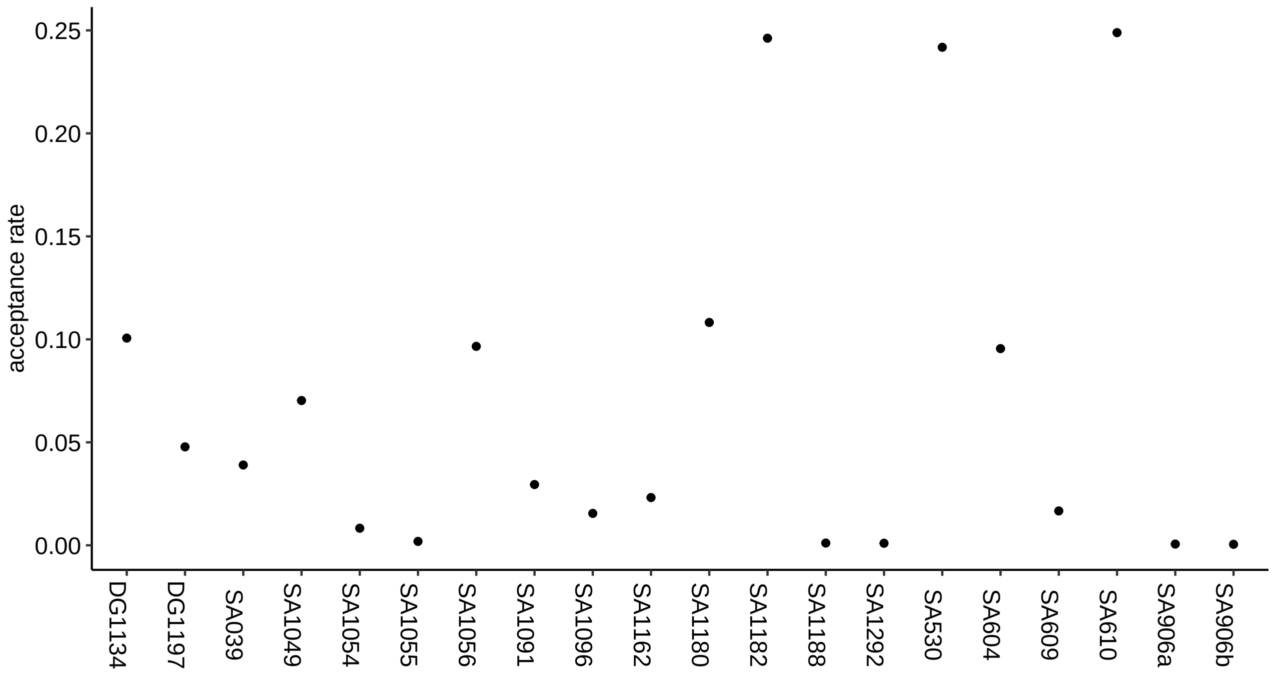

Supplementary Fig. 25: **Acceptance rate of approximate Bayesian computation sequential Monte Carlo (ABC SMC) applied to 20 single-cell data whole-genome sequencing datasets.** Source data are provided as a Source Data file.

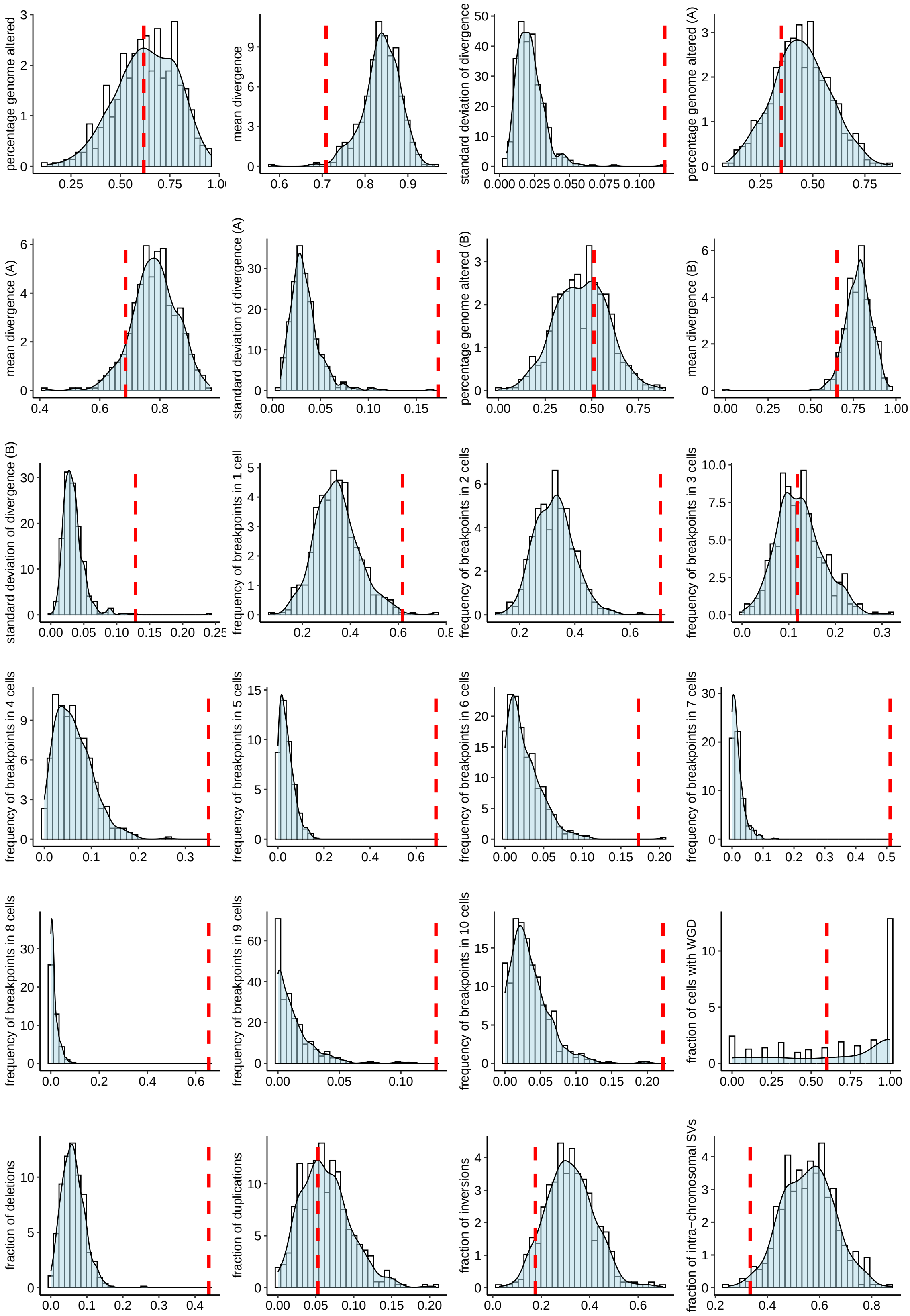

Supplementary Fig. 26: **Posterior predictive distributions of the summary statistics used for inference from a single-cell whole-genome sequencing dataset SA1055.** The red dashed line indicates the observed value. The observed values for 15 out of the 24 summary statistics fell within two standard deviations of the corresponding distributions. Each plot contains 500 data points, which represent the posterior samples. Source data are provided as a Source Data file.

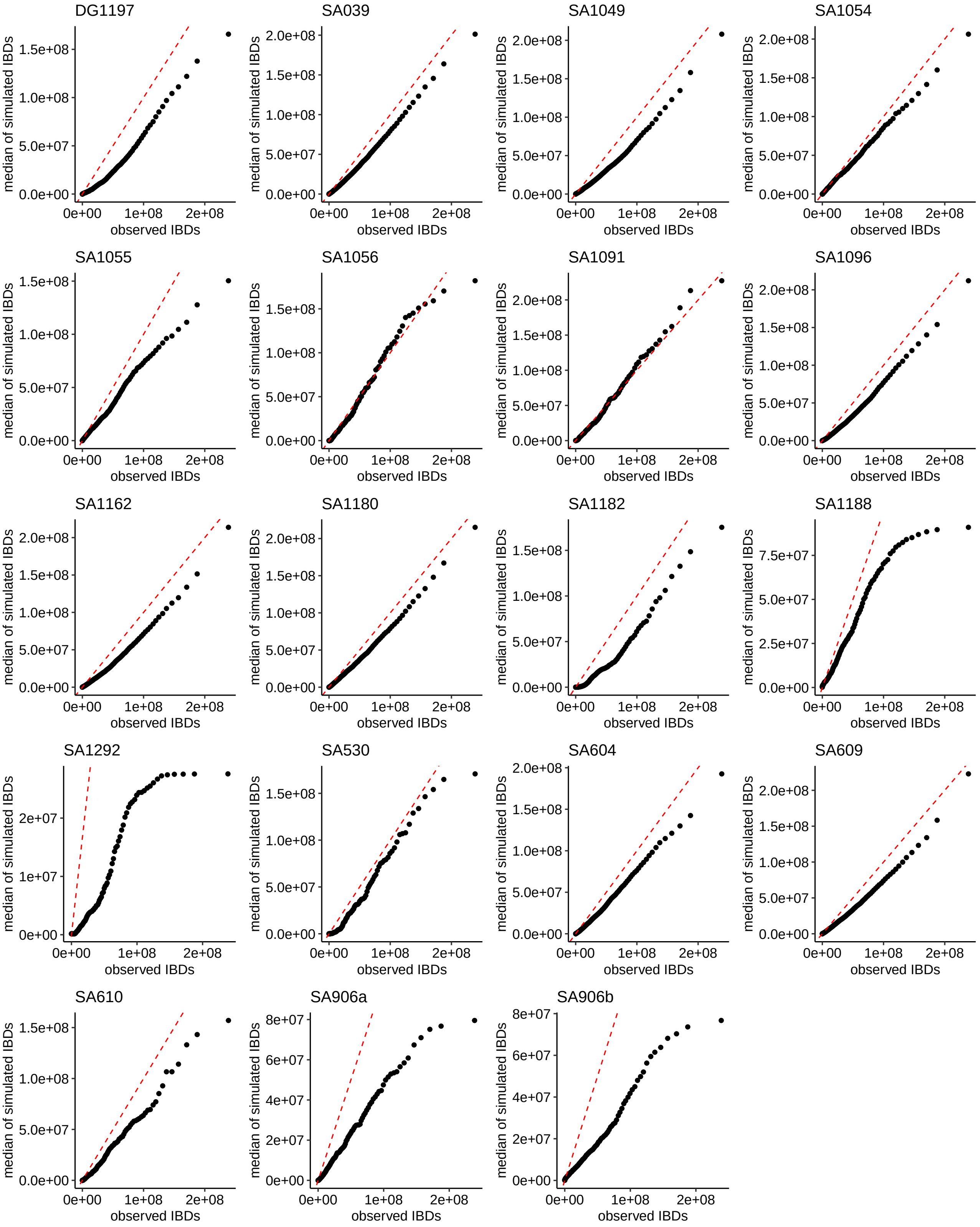

Supplementary Fig. 27: **Quantile-Quantile (QQ) plots comparing the simulated and real distributions of inter-breakpoint distances (IBDs) across 19 well-fit single-cell whole-genome sequencing datasets.** The simulated data were generated using parameters sampled from the posterior distributions of the inferred parameters. The red dashed line indicates $y = x$. Source data are provided as a Source Data file.

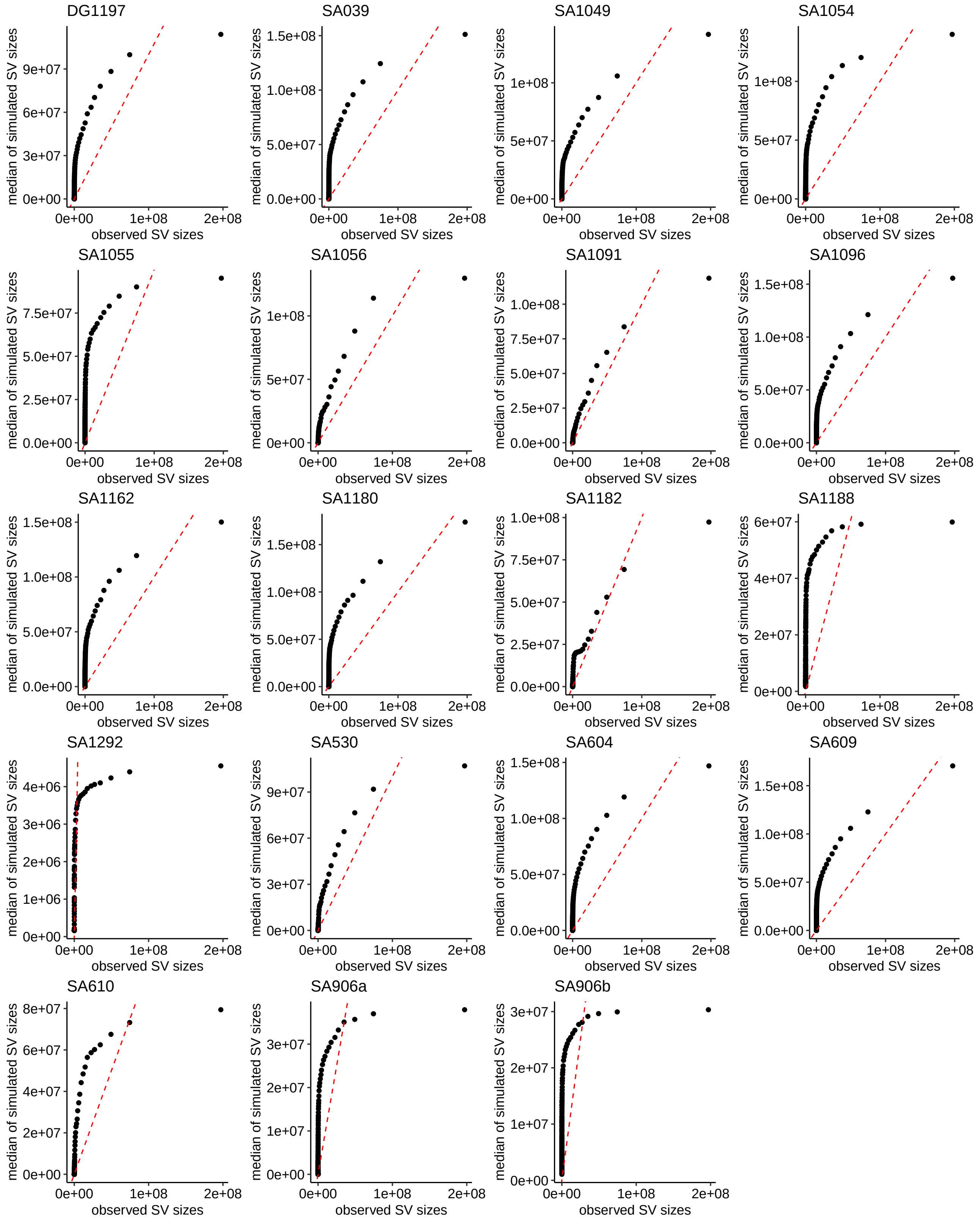

Supplementary Fig. 28: **Quantile-Quantile (QQ) plots comparing the simulated and real distributions of structural variant (SV) sizes across 19 well-fit single-cell whole-genome sequencing datasets.** The simulated data were generated using parameters sampled from the posterior distributions of the inferred parameters. The red dashed line indicates $y = x$. Source data are provided as a Source Data file.

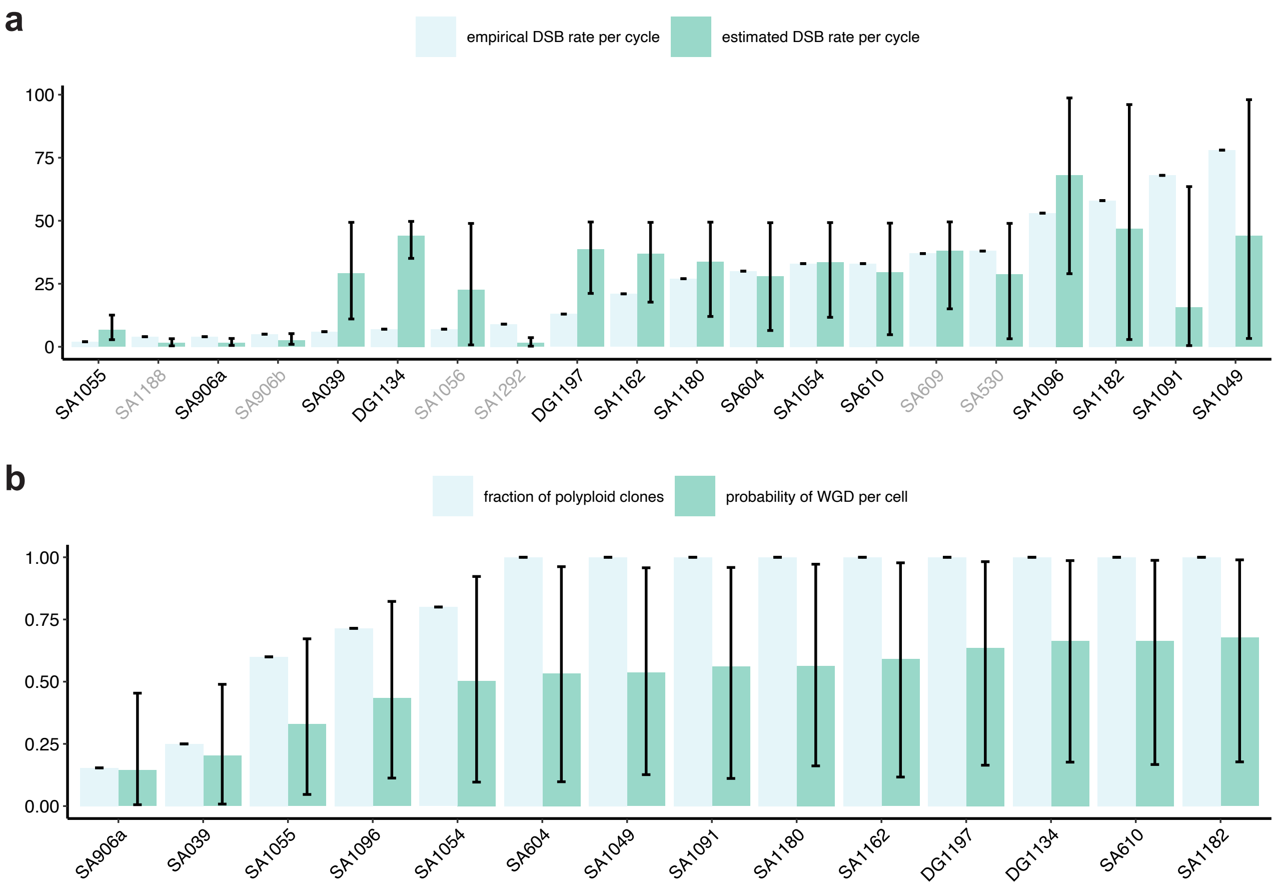

Supplementary Fig. 29: **Comparison of estimated parameters with empirical values across 20 single-cell whole-genome sequencing datasets. a,** The comparison of estimated and empirical double-strand break (DSB) rate per cycle. **b,** The comparison of estimated probability of whole genome doubling (WGD) per cell and calculated fraction of polyploid clones in each dataset. The error bar for the estimated parameter represents the 95% credible interval of the posterior mean, calculated from 500 posterior samples. There are 14 datasets with WGD. The names of datasets without WGD are shown in grey. Source data are provided as a Source Data file.

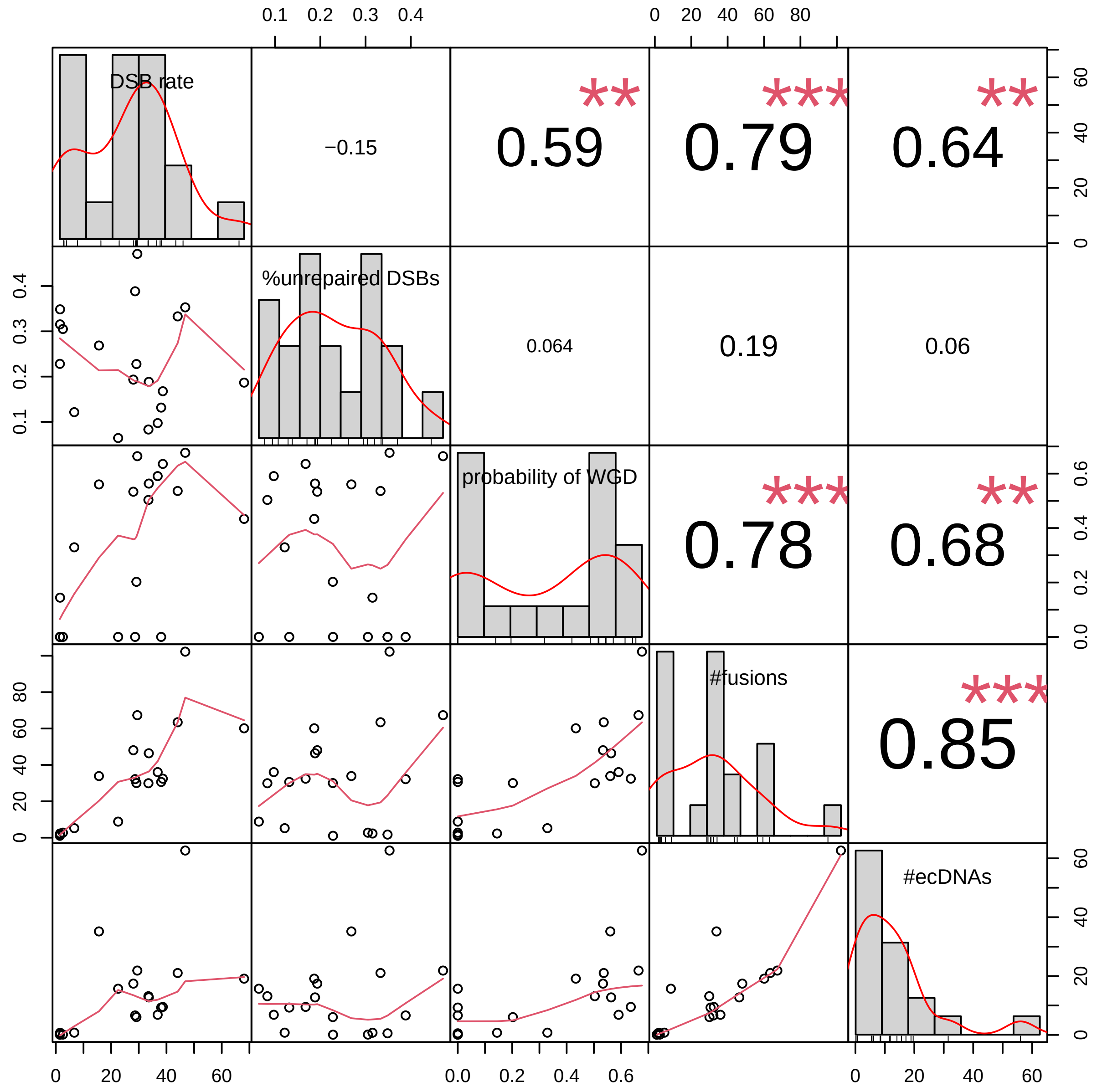

Supplementary Fig. 30: **Pairwise correlations of parameters inferred from 19 well-fit single-cell whole-genome sequencing datasets.** The parameters include the inferred double-strand break (DSB) rate per cycle, fraction of unrepaired DSBs per cycle, probability of whole genome doubling (WGD) per cell, mean number of chromosome fusions per cycle, and mean number of extrachromosomal circular DNAs (ecDNAs) per cell. The Spearman correlation coefficient and corresponding two-sided p-value are shown for each pair of parameters. The indicator of significance: * – p-val < 0.05, ** – p-val < 0.01, *** – p-val < 0.001. Source data are provided as a Source Data file.

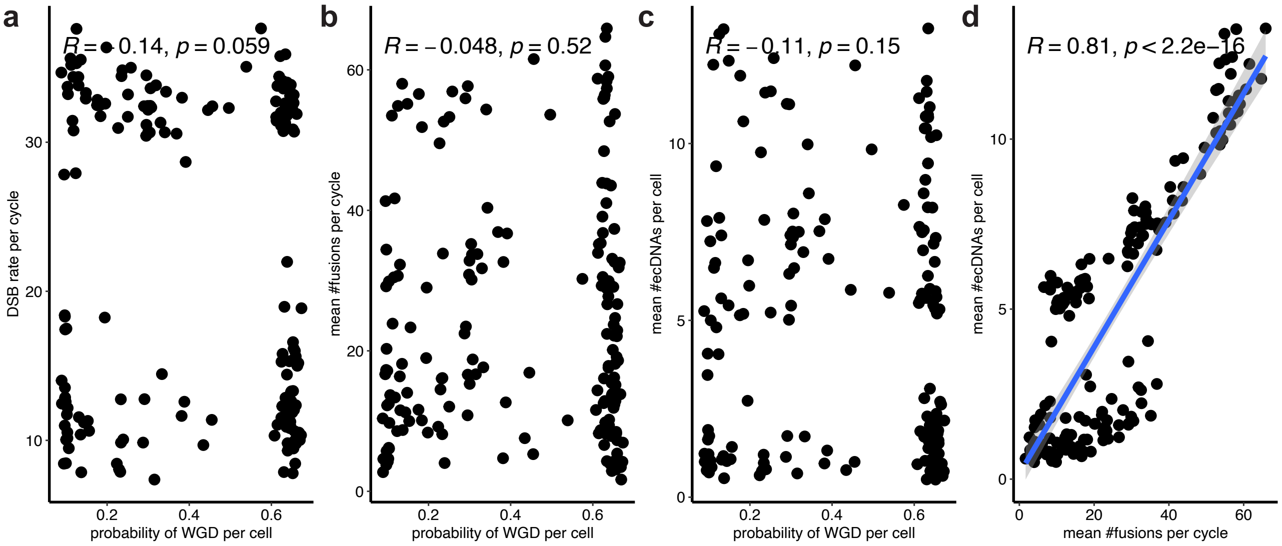

Supplementary Fig. 31: **Pairwise correlations of parameters inferred from the simulated data**. **a,** The correlation between the inferred probability of whole genome doubling (WGD) per cell and double-strand break (DSB) rate per cycle. **b,** The correlation between the inferred mean number of chromosome fusions per cycle and probability of WGD per cell. **c,** The correlation between the inferred mean number of extrachromosomal circular DNAs (ecDNAs) per cell and probability of WGD per cell. **d,** The correlation between the inferred mean number of ecDNAs per cell and mean number of chromosome fusions per cycle. The Spearman correlation coefficient and corresponding two-sided p-value are shown for each plot. The shaded area shows the 95% confidence interval of linear regression. The plots correspond to the same simulation data presented in Supplementary Fig. 21. There are 180 data points on each plot, where the same set of parameters generated 10 data points. Source data are provided as a Source Data file.

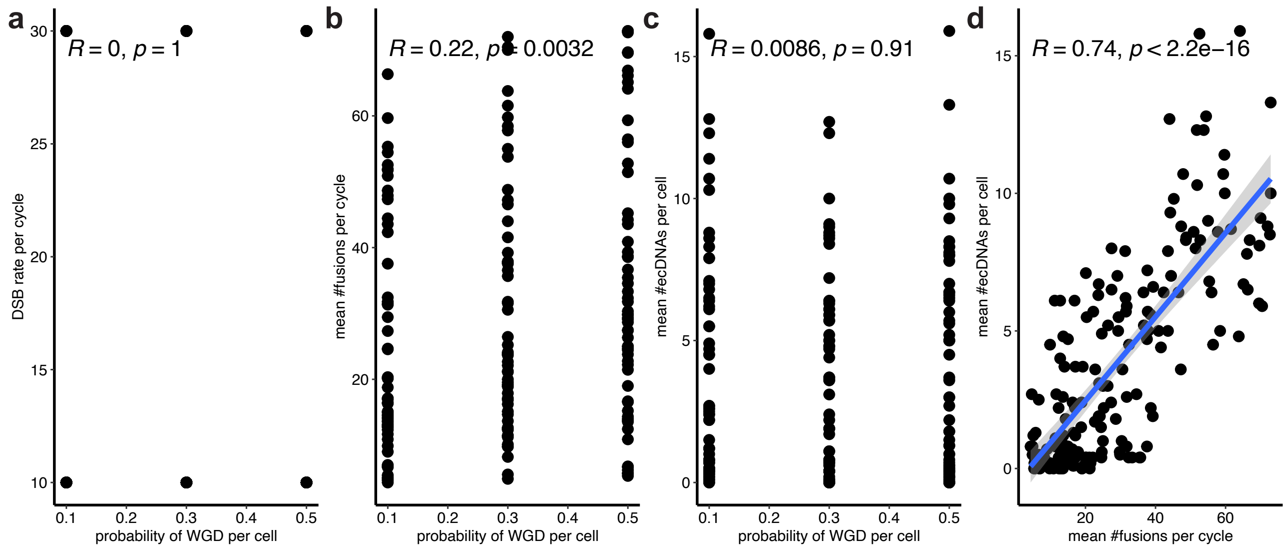

Supplementary Fig. 32: **Pairwise correlations of real parameters in the simulated data**. **a,** The correlation between real probability of whole genome doubling (WGD) per cell and double-strand break (DSB) rate per cycle. **b,** The correlation between real mean number of chromosome fusions per cycle and probability of WGD per cell. **c,** The correlation between real mean number of extrachromosomal circular DNAs (ecDNAs) per cell and probability of WGD per cell. **d,** The correlation between real mean number of ecDNAs per cell and mean number of chromosome fusions per cycle. The Spearman correlation coefficient and corresponding two-sided p-value are shown for each plot. The shaded area shows the 95% confidence interval of linear regression. The plots correspond to the same simulation data presented in Supplementary Fig. 21. There are 180 data points on each plot, where the same set of parameters generated 10 data points. Source data are provided as a Source Data file.

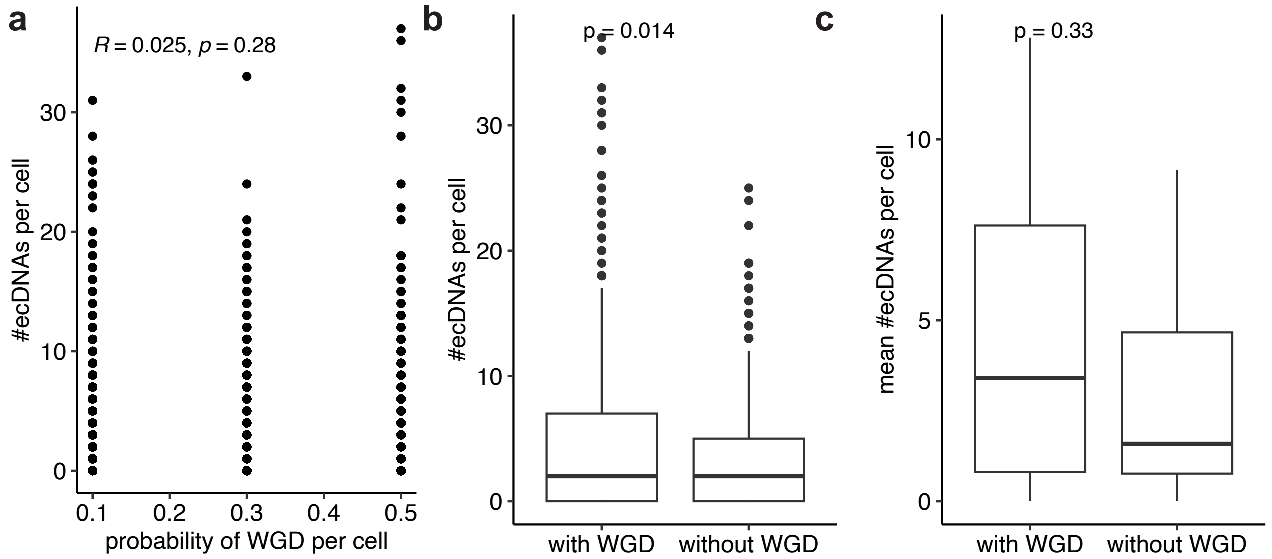

Supplementary Fig. 33: **Relationships between real number of extrachromosomal circular DNAs (ecDNAs) and whole genome doubling (WGD) in the simulated data**. **a,** The relationship between real number of ecDNAs per cell and probability of WGD per cell. The Spearman correlation coefficient and corresponding two-sided p-value are shown. **b,** The number of ecDNAs per cell in cells with and without WGD. **c,** The mean number of ecDNAs per cell in cells with and without WGD.The plots correspond to the same simulation data presented in Supplementary Fig. 21. There are 180 simulated datasets, each consisting of 10 cells, with 10 replicates for each of the 18 parameter settings. Among the 1,800 cells, 600 cells correspond to each specific probability of WGD in **a**. In **b** and **c**, 1,134 cells exhibit WGD, while the remaining 666 cells do not. Two-sided Wilcoxon tests were used to compute p-values in **b** and **c**. The box plots show the median (centre), 1st (lower hinge), and 3rd (upper hinge) quartiles of the data; the whiskers extend to 1.5 times of the interquartile range (distance between the 1st and 3rd quartiles); data beyond the interquartile range are plotted individually. Source data are provided as a Source Data file.

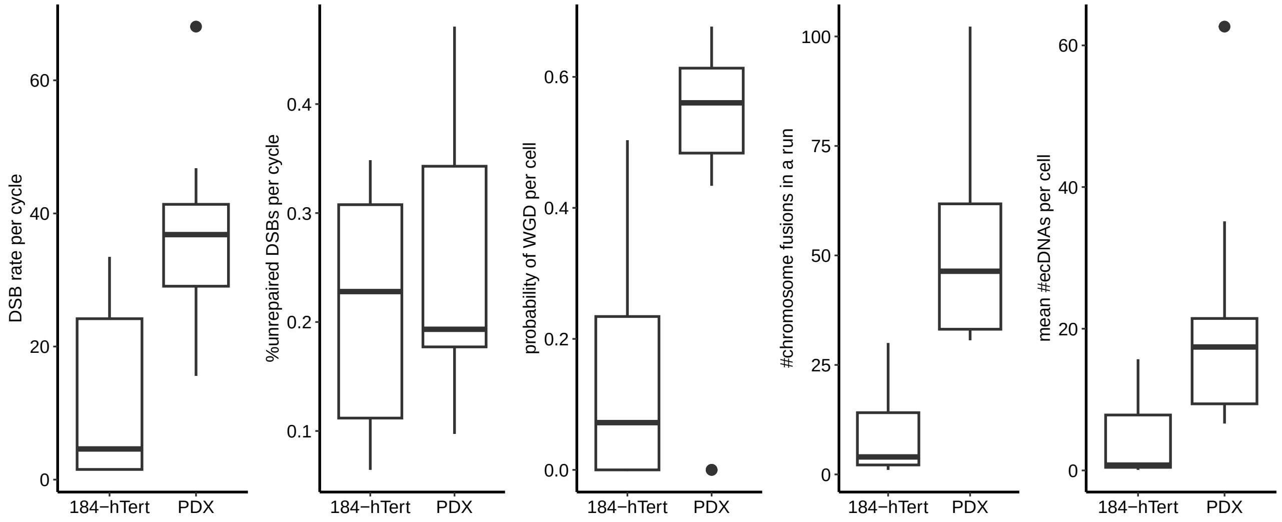

Supplementary Fig. 34: **Comparison of posterior means of parameters estimated from 19 well-fit single-cell whole-genome sequencing datasets of different cell types.** The box plots show the median (centre), 1st (lower hinge), and 3rd (upper hinge) quartiles of the data; the whiskers extend to 1.5 times of the interquartile range (distance between the 1st and 3rd quartiles); data beyond the interquartile range are plotted individually. There are 8 184-hTert mammary epithelial cell line datasets and 11 patient-derived xenograft (PDX) datasets. Source data are provided as a Source Data file.

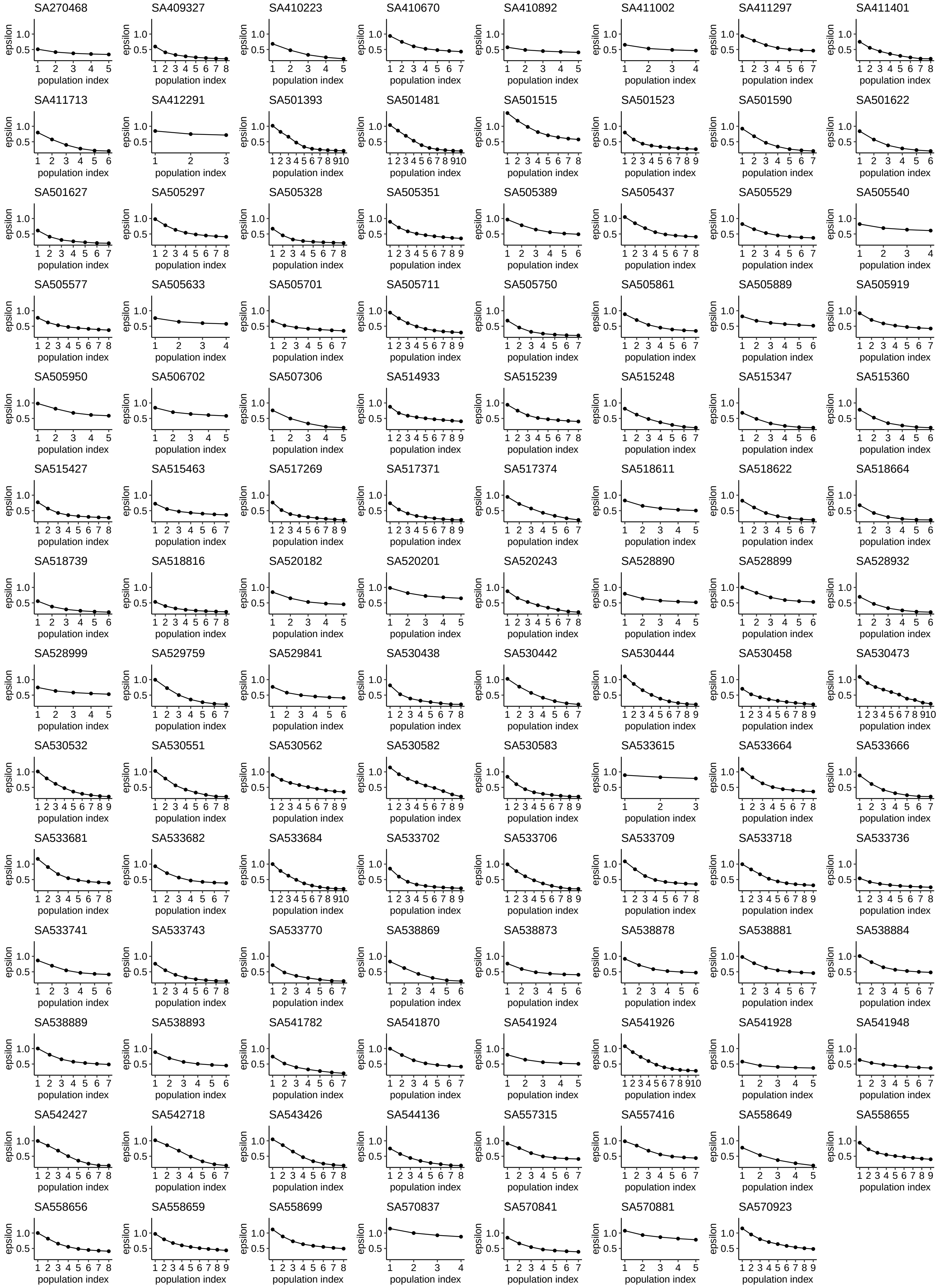

Supplementary Fig. 35: **Tolerance change across populations of approximate Bayesian computation sequential Monte Carlo (ABC SMC) applied to 111 bulk whole-genome sequencing datasets.** Source data are provided as a Source Data file.

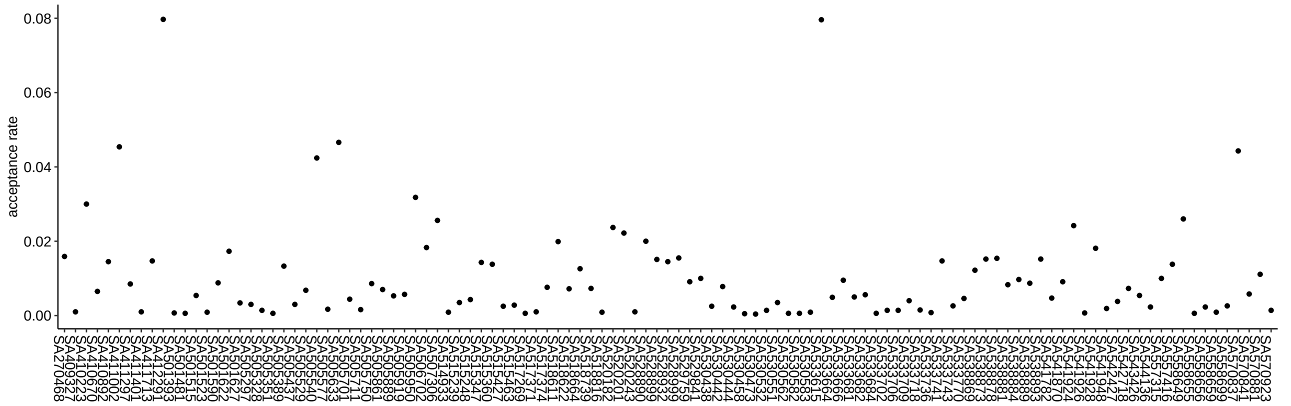

Supplementary Fig. 36: **Acceptance rate of approximate Bayesian computation sequential Monte Carlo (ABC SMC) applied to 111 bulk whole-genome sequencing datasets.** Source data are provided as a Source Data file.

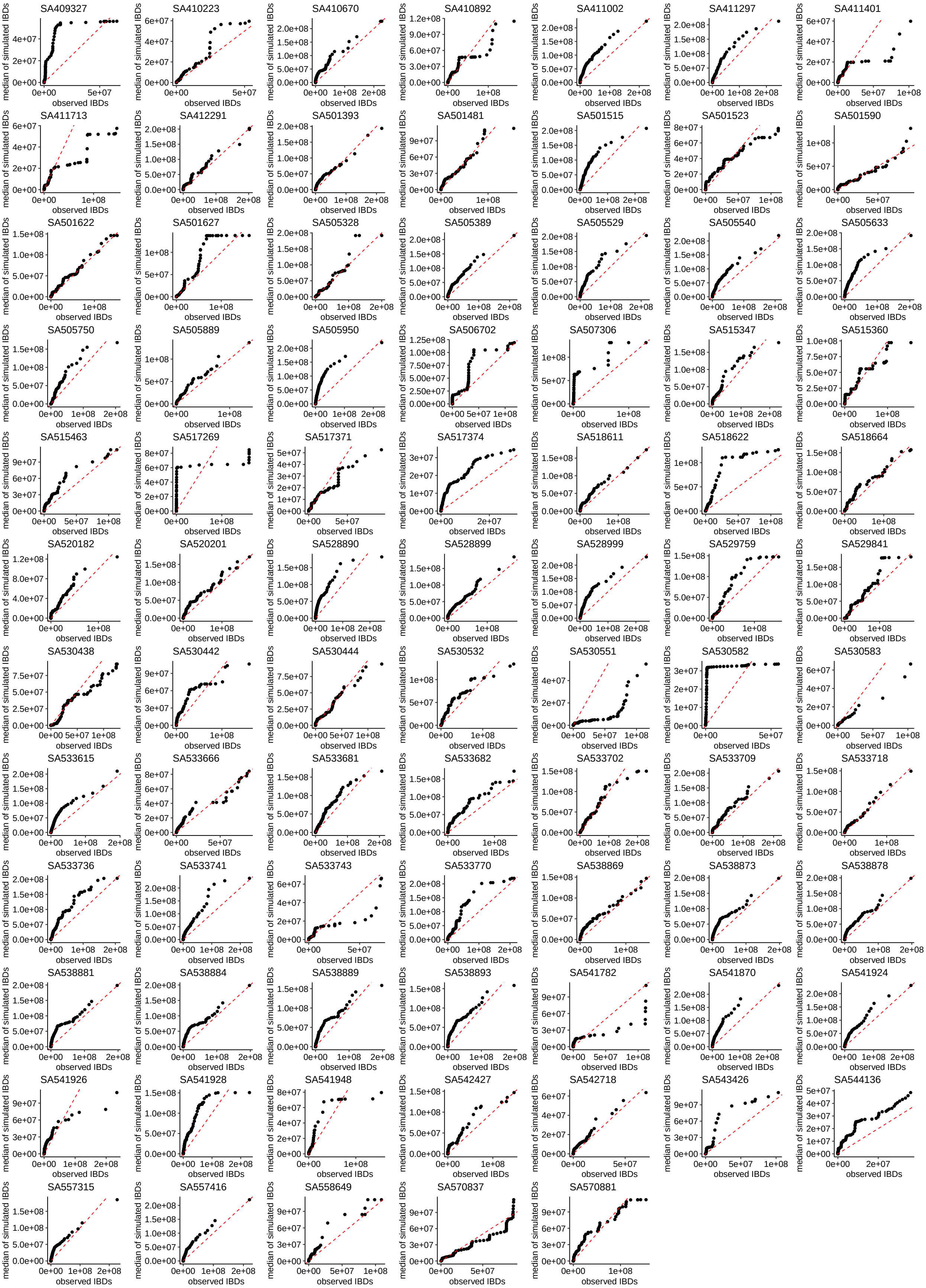

Supplementary Fig. 37: **Quantile-Quantile (QQ) plots comparing the simulated and real distributions of inter-breakpoint distances (IBDs) across 82 well-fit bulk whole-genome sequencing datasets.** The simulated data were generated using parameters sampled from the posterior distributions of the inferred parameters. The red dashed line indicates $y = x$. Source data are provided as a Source Data file.

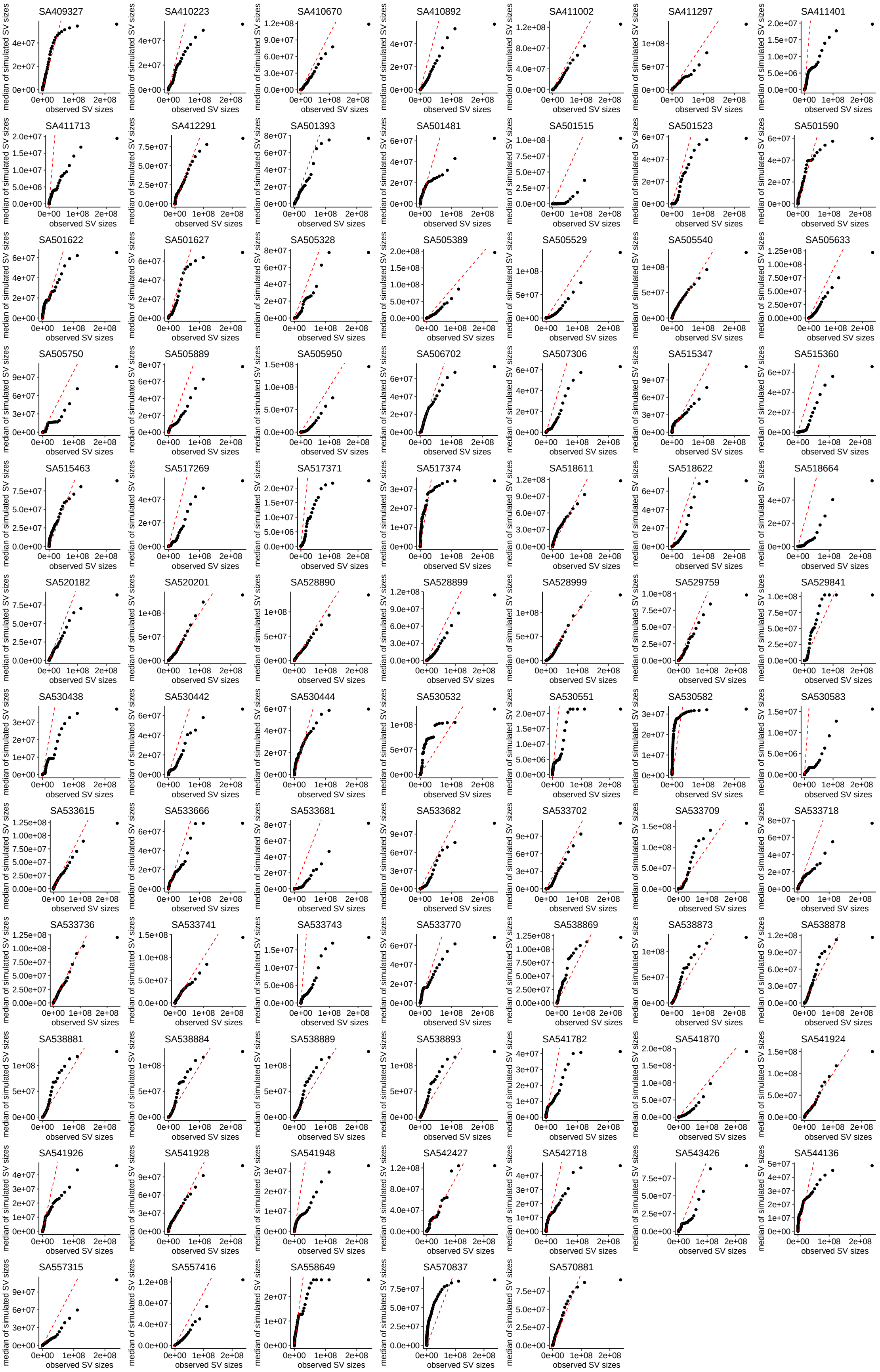

Supplementary Fig. 38: **Quantile-Quantile (QQ) plots comparing the simulated and real distributions of structural variant (SV) sizes across 82 well-fit bulk whole-genome sequencing datasets.** The simulated data were generated using parameters sampled from the posterior distributions of the inferred parameters. The red dashed line indicates $y = x$. Source data are provided as a Source Data file.

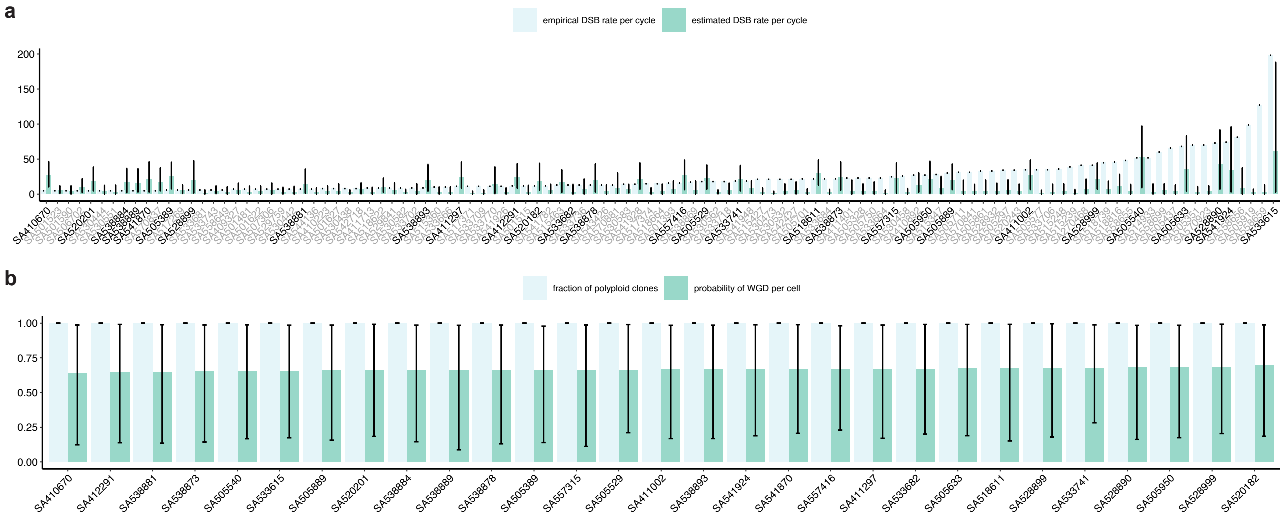

Supplementary Fig. 39: **Comparison of estimated parameters with empirical values across 111 bulk whole-genome sequencing datasets. a,** The comparison of estimated and empirical double-strand break (DSB) rate per cycle. **b,** The comparison of estimated probability of whole genome doubling (WGD) per cell and calculated fraction of polyploid clones in each dataset. The error bar for the estimated parameter represents the 95% credible interval of the posterior mean, calculated from 500 posterior samples. There are 29 datasets with WGD. The names of datasets without WGD are shown in grey. Source data are provided as a Source Data file.

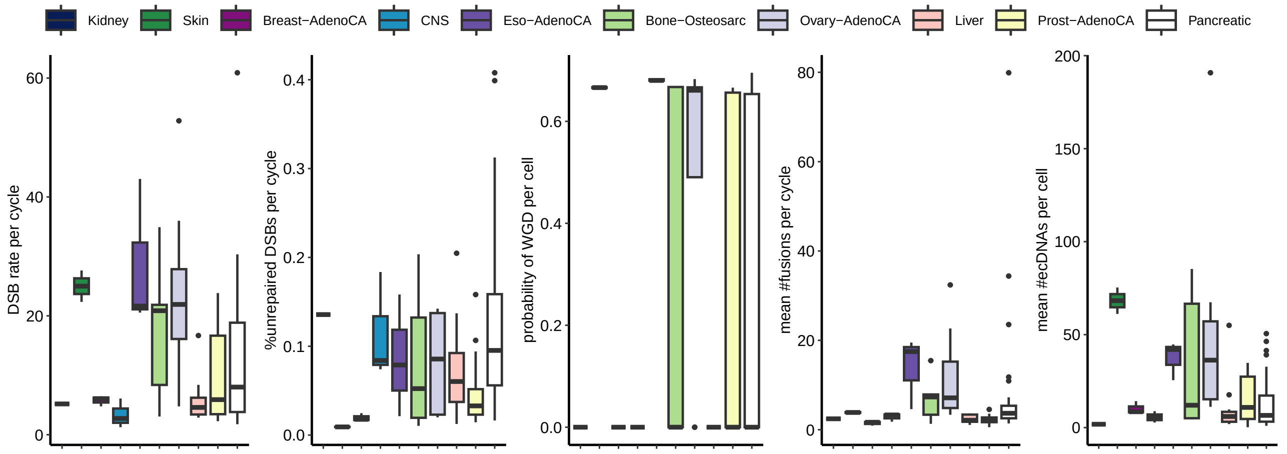

Supplementary Fig. 40: **Comparison of posterior means of parameters estimated from 82 well-fit bulk whole-genome sequencing datasets across cancer types.** The box plots show the median (centre), 1st (lower hinge), and 3rd (upper hinge) quartiles of the data; the whiskers extend to 1.5 times of the interquartile range (distance between the 1st and 3rd quartiles); data beyond the interquartile range are plotted individually. There are 1 kidney cancer dataset, 2 skin cancer dataset, 3 breast adenocarcinoma datasets, 3 central nervous system (CNS) cancer datasets, 3 esophageal adenocarcinoma datasets, 5 bone osteosarcoma datasets, 8 ovary adenocarcinoma datasets, 12 liver cancer datasets, 15 prostate adenocarcinoma datasets, and 30 pancreatic cancer datasets. Source data are provided as a Source Data file.

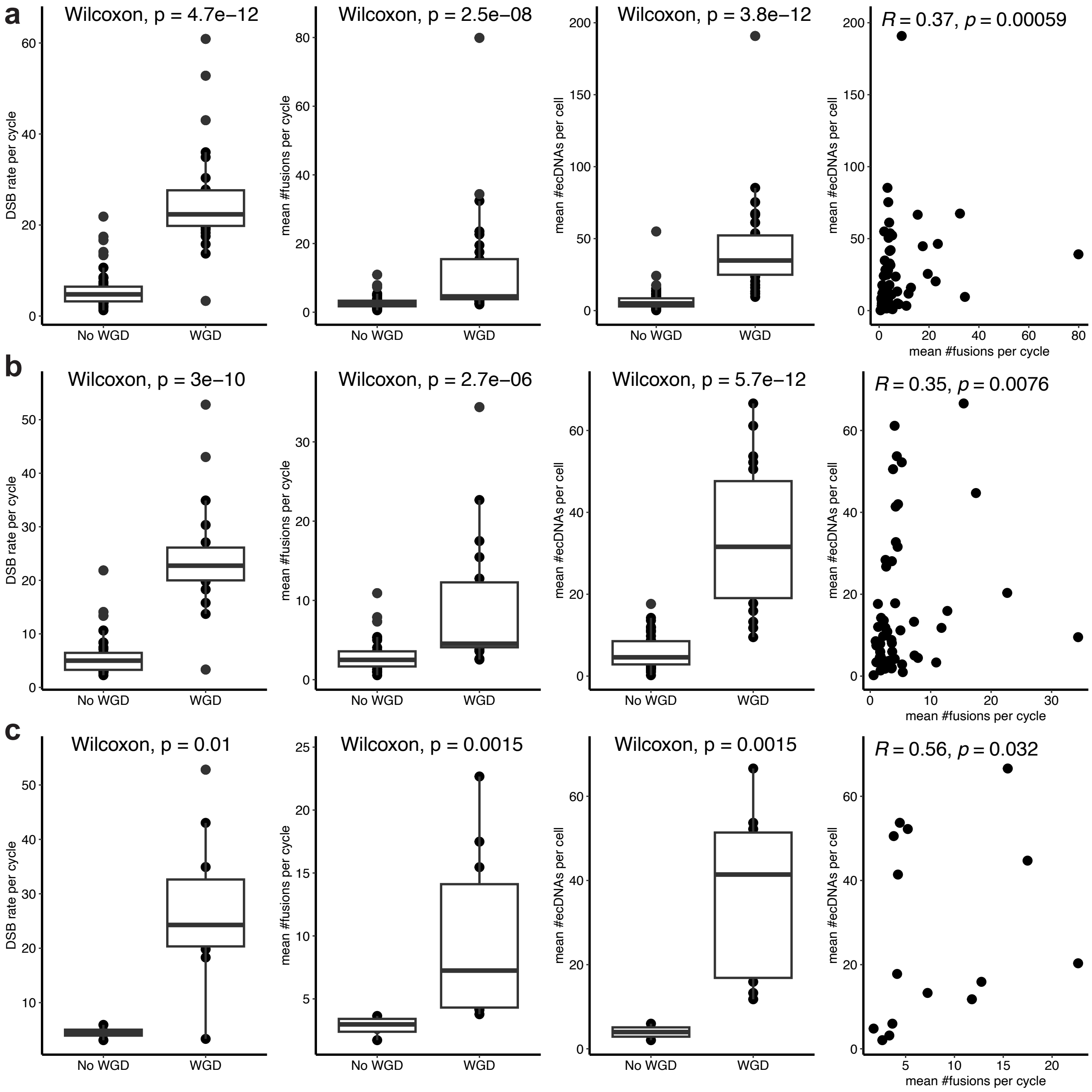

Supplementary Fig. 41: **Pairwise relationships of parameters inferred from bulk whole-genome sequencing datasets. a,** The relationships between parameters inferred from 82 well-fit datasets. There are 53 datasets without whole genome doubling (WGD) and 29 datasets with WGD. **b,** The relationships between parameters inferred from 56 well-fit datasets with inversion enrichment (fraction exceeding 25%). There are 37 datasets without WGD and 19 datasets with WGD. **c,** The relationships between parameters inferred from 15 datasets with both *CCNE1* amplifications and inversion enrichment. There are 4 datasets without WGD and 11 datasets with WGD. Since datasets have either WGD or no WGD and the inferred probabilities of WGD were centered around 0.6, Wilcoxon rank-sum test was used to compare differences in the inferred parameters between samples with and without WGD. For plots in the last column, the Spearman correlation coefficients and corresponding two-sided p-values are shown. The box plots show the median (centre), 1st (lower hinge), and 3rd (upper hinge) quartiles of the data; the whiskers extend to 1.5 times of the interquartile range (distance between the 1st and 3rd quartiles); data beyond the interquartile range are plotted individually. Source data are provided as a Source Data file.

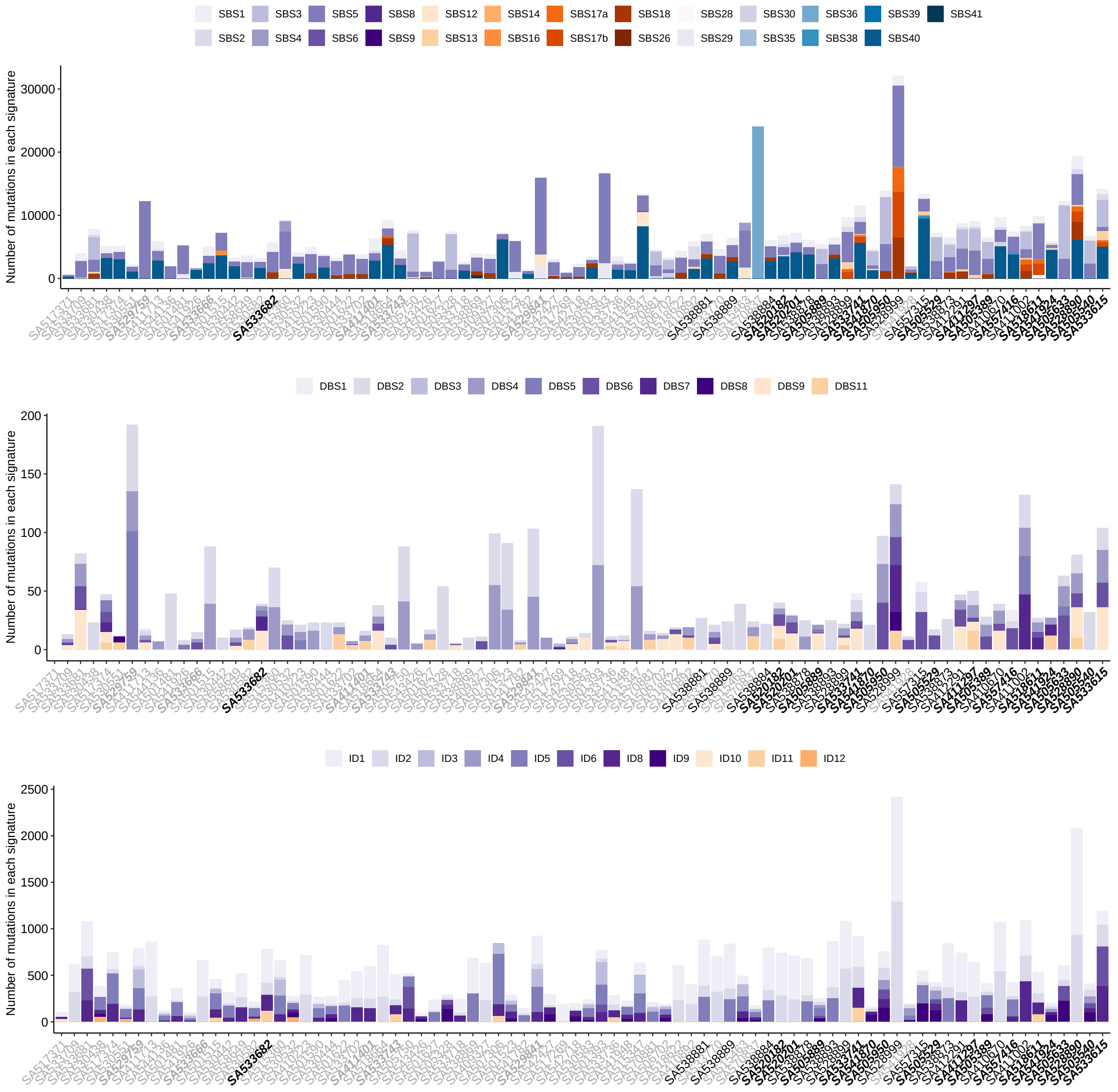

Supplementary Fig. 42: **Distribution of mutational signatures across 82 well-fit bulk whole-genome sequencing datasets.** The mutational signatures were extracted from the known signatures generated by SigProfiler from the Pan-Cancer Analysis of Whole Genomes (PCAWG) Consortium. SBS: single-base substitution, DBS: doublet-base substitution, ID: indel. Source data are provided as a Source Data file.

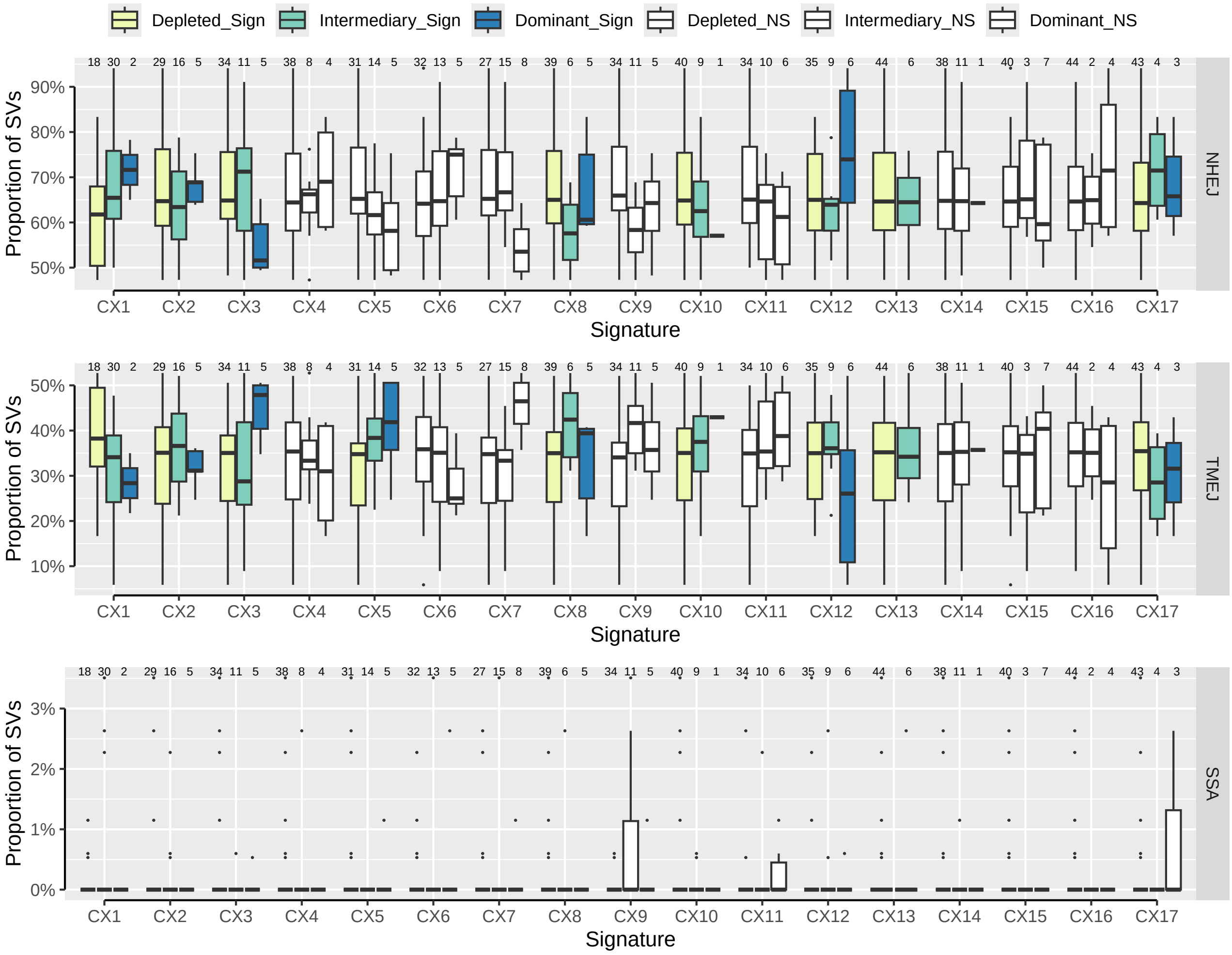

Supplementary Fig. 43: **Association between copy number signature activities and structural variants (SVs) across 50 well-fit bulk whole-genome sequencing datasets.** Based on sequence microhomogies, SVs were categorized according to the most likely repair pathways: NHEJ (non-homologous end joining), TMEJ (polymerase theta-mediated end joining), and SSA (single strand annealing). Specifically, SVs with 0-1 bp microhomologies were assigned to the NHEJ pathway, those with 2-20 bp to the TMEJ pathway, and SVs with longer microhomologies were categorized into the SSA pathway. Based on signature activity values, the 50 datasets are grouped into three categories for each signature: depleted (activity < 0), intermediary (0 <= activity <= 1.25), and dominant (activity > 1.25). The box plots show the median (centre), 1st (lower hinge), and 3rd (upper hinge) quartiles of the data; the whiskers extend to 1.5 times of the interquartile range (distance between the 1st and 3rd quartiles); data beyond the interquartile range are plotted individually. The number above each box plot represents the sample size. For each dataset, the proportions of SVs across different pathways for a given copy number signature sum up to 1. Statistical significance was assessed using two-sided Welch's t-test. “_Sign” means statistically significant and “_NS” means not statistically significant. Source data are provided as a Source Data file.

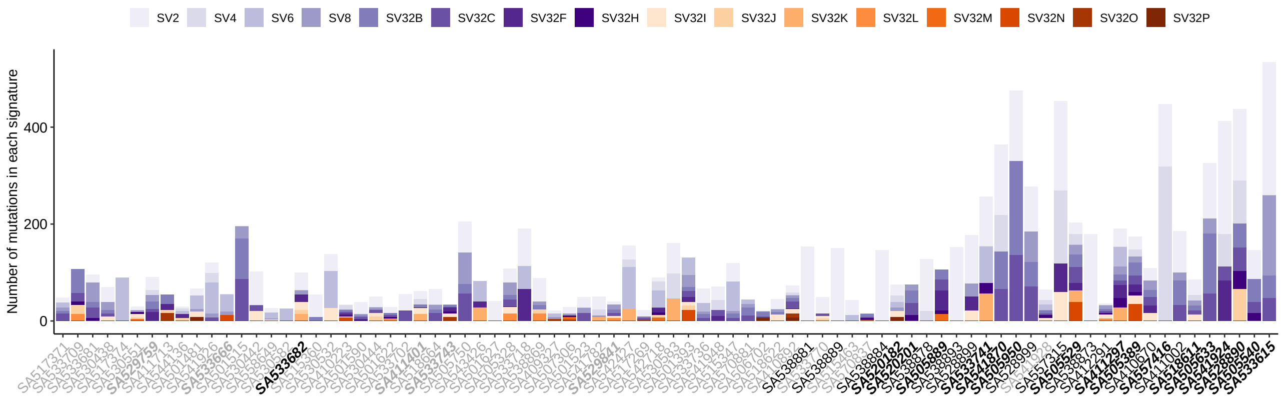

Supplementary Fig. 44: **Distribution of structural variant (SV) signatures across 82 well-fit bulk whole-genome sequencing datasets.** The SV signatures were firstly generated by SigProfiler using 1,815 PCAWG samples with SVs, and the signatures for the 82 samples were subsequently extracted. Source data are provided as a Source Data file.

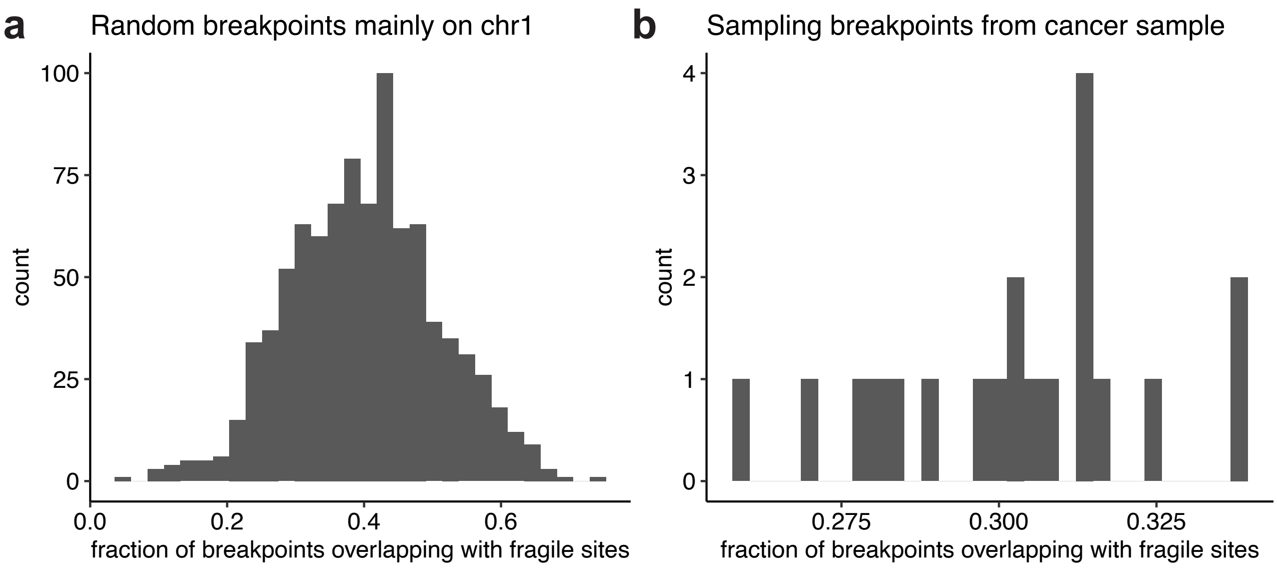

Supplementary Fig. 45: **Fractions of breakpoints overlapping with fragile sites in the simulated data. a,** The distribution of breakpoints overlapping with fragile sites when random breakpoints were introduced primarily on chr1 to generate the simulated "true" data for the results presented in Fig. 3b-e. There are 900 datasets, generated from 50 simulations for each of the 18 different parameter settings. **b,** The distribution of breakpoints overlapping with fragile sites when breakpoints were sampled from a primary breast cancer patient. The data were simulated using the same parameters as those in Fig. 5b-e. There are 20 datasets, generated from 10 simulations for each of the two different parameter settings. Source data are provided as a Source Data file.
