# Supplementary material for: Cell-cycle dependent DNA repair and replication unifies patterns of chromosome instability
Source: Nat Commun. 2025 Mar 28;16:3033. doi: 10.1038/s41467-025-58245-z (PMC11953314; doi:10.1038/s41467-025-58245-z)
Supplement: Supplementary file 2 — Reporting Summary [file 41467_2025_58245_MOESM2_ESM.pdf]

nature portfolio

Corresponding author(s): Chris P. Barnes
Bingxin Lu

Last updated by author(s): Mar 6, 2025

Reporting Summary

Nature Portfolio wishes to improve the reproducibility of the work that we publish. This form provides structure for consistency and transparency in reporting. For further information on Nature Portfolio policies, see our Editorial Policies and the Editorial Policy Checklist.

Statistics

For all statistical analyses, confirm that the following items are present in the figure legend, table legend, main text, or Methods section.

n/a	Confirmed	
☐	☒	The exact sample size (*n*) for each experimental group/condition, given as a discrete number and unit of measurement
☐	☒	A statement on whether measurements were taken from distinct samples or whether the same sample was measured repeatedly
☐	☒	The statistical test(s) used AND whether they are one- or two-sided<br>*Only common tests should be described solely by name; describe more complex techniques in the Methods section.*
☐	☒	A description of all covariates tested
☐	☒	A description of any assumptions or corrections, such as tests of normality and adjustment for multiple comparisons
☐	☒	A full description of the statistical parameters including central tendency (e.g. means) or other basic estimates (e.g. regression coefficient) AND variation (e.g. standard deviation) or associated estimates of uncertainty (e.g. confidence intervals)
☐	☒	For null hypothesis testing, the test statistic (e.g. *F*, *t*, *r*) with confidence intervals, effect sizes, degrees of freedom and *P* value noted<br>*Give P values as exact values whenever suitable.*
☐	☒	For Bayesian analysis, information on the choice of priors and Markov chain Monte Carlo settings
☒	☐	For hierarchical and complex designs, identification of the appropriate level for tests and full reporting of outcomes
☒	☐	Estimates of effect sizes (e.g. Cohen's *d*, Pearson's *r*), indicating how they were calculated

Our web collection on statistics for biologists contains articles on many of the points above.

Software and code

Policy information about availability of computer code

Data collection	No software was used for data collection.<br>The initially processed single-cell whole-genome sequencing data were obtained from the published paper (https://doi.org/10.1038/s41586-022-05249-0).<br>The initially processed bulk whole-genome sequencing data were obtained from the International Cancer Genome Consortium Accelerating Research in Genomic Oncology (ICGC ARGO) data platform (https://platform.icgc-argo.org/).<br>The cancer-related genes and sample breakpoints used for simulating data during validation of parameter inference were obtained from the Catalogue Of Somatic Mutations In Cancer (COSMIC) database (https://cancer.sanger.ac.uk/cosmic)..

Data analysis

The algorithm of the reported computational model was developed in C++11 and the data analysis scripts were written in R and Bash.
The code is available at https://github.com/ucl-cssb/CIN_SV.
The scripts were run with R 4.X and Bash 4.2.46.

External packages used:
ShatterSeek v1.1 for detecting chromothripsis (https://github.com/parklab/ShatterSeek).
Jabba v1.1 for detecting chromoplexy (https://github.com/mskilab-org/JaBbA).
SeismicAmplification for detecting seismic amplification (https://github.com/seismicon/SeismicAmplification).
ApproxBayes v0.3.2 for running ABC SMC (https://github.com/marcjwilliams1/ApproxBayes.jl).
SigProfiler v1.1.24 for detecting structural variant signature (https://github.com/AlexandrovLab/SigProfilerExtractor).

For manuscripts utilizing custom algorithms or software that are central to the research but not yet described in published literature, software must be made available to editors and reviewers. We strongly encourage code deposition in a community repository (e.g. GitHub). See the Nature Portfolio guidelines for submitting code & software for further information.

Data

Policy information about availability of data

All manuscripts must include a data availability statement. This statement should provide the following information, where applicable:

- Accession codes, unique identifiers, or web links for publicly available datasets
- A description of any restrictions on data availability
- For clinical datasets or third party data, please ensure that the statement adheres to our policy

The simulated data and processed single-cell and bulk whole-genome sequencing data generated in this study have been deposited in Zenodo [72]. The initially processed single-cell whole-genome sequencing data used in this study are available at Zenodo (https://zenodo.org/record/6998936). The initially processed bulk whole-genome sequencing data used in this study are available at the International Cancer Genome Consortium Accelerating Research in Genomic Oncology (ICGC ARGO) data platform (https://platform.icgc-argo.org/). The breakpoints used for simulating data during validation of parameter inference, as well as the cancer-related genes, were obtained from the Catalogue Of Somatic Mutations In Cancer (COSMIC) database (https://cancer.sanger.ac.uk/cosmic). The ecDNAs from the eccDNAdb database were obtained from Supplementary Table S2 of the corresponding paper [56]. Source data are provided with this paper.

Research involving human participants, their data, or biological material

Policy information about studies with human participants or human data. See also policy information about sex, gender (identity/presentation), and sexual orientation and race, ethnicity and racism.

Reporting on sex and gender	This study did not involve human participants directly, because we used processed data from mammary epithelial cell lines, patient-derived xenografts, and patient samples.
Reporting on race, ethnicity, or other socially relevant groupings	Not applicable.
Population characteristics	Not applicable.
Recruitment	Not applicable.
Ethics oversight	Not applicable.

Note that full information on the approval of the study protocol must also be provided in the manuscript.

Field-specific reporting

Please select the one below that is the best fit for your research. If you are not sure, read the appropriate sections before making your selection.

[x] Life sciences [ ] Behavioural & social sciences [ ] Ecological, evolutionary & environmental sciences

For a reference copy of the document with all sections, see nature.com/documents/nr-reporting-summary-flat.pdf

Life sciences study design

All studies must disclose on these points even when the disclosure is negative.

Sample size

We used 20 processed single-cell whole-genome sequencing datasets, including 8 datasets from 184-hTERT mammary epithelial cell lines and 12 datasets from FBI (fold back inversions) tumours in patient-derived xenografts (PDXs) of primary cancer patients for inference with approximate Bayesian computation sequential Monte Carlo (ABC SMC). These datasets contain both copy number alterations (CNAs) and structural variants (SVs), making them well-suited for applying our model.
We also used all 1,815 processed bulk whole-genome sequencing datasets with SVs from the Pan-Cancer Analysis of Whole Genomes (PCAWG) Consortium for analysis. These datasets have been comprehensively analyzed, including the detection of CNAs, SVs, extrachromosomal circular DNAs (ecDNAs), breakage-fusion-bridges (BFBs), and chromothripsis, making them ideal for validating our model at a larger scale. To facilitate validation, we selected PCAWG samples with at least three clones and the presence of CNAs, SVs, ecDNAs, BFBs for

	inference with ABC SMC, and chromothripsis, including 148 samples without WGD and 5 samples with WGD. To examine more samples with WGD, we also included 27 additional samples that may not have complex SVs detected. Of these 180 samples, 111 PCAWG samples successfully completed ABC SMC, with 29 exhibiting WGD.
Data exclusions	We excluded 11 PDX datasets belonging to HRD-Dup (homologous recombination deficiency-duplication) and TD (tandem duplication) tumours, because our model is not suitable for structural variants which were probably generated from HRD or other replication errors like TD.<br>We excluded PCAWG samples with fewer than 10 SVs or an empirical DSB rate below five to ensure sufficient data for inference. We excluded SVs without clonal assignments and computed breakpoint frequency distributions based on the known probabilistic assignment of each SV to the detected clones.
Replication	We validated our approach through repeated simulations using different random seeds under the same parameters.<br>To examine the relationship between chromothripsis and ecDNAs, we repeated 50 times the simulation shown in Supplementary Fig. 1 (Supplementary Fig. 4).<br>To explore the interaction among WGD, chromothripsis, and ecDNAs, we repeated 50 times the simulations shown in Supplementary Table 3 when the probability of WGD was 0.1 (Supplementary Fig. 5) and 0.122 (Supplementary Fig. 6), respectively.<br>To determine the minimum number of misrepaired double-strand breaks (DSBs) required to generate chromoplexy, we conducted simulations with progressively fewer misrepaired DSBs per cycle (5, 2, and 1) on random chromosomes in each cell cycle, with 10 repeats for each parameter setting (Supplementary Fig. 9).<br>To investigate the formation of chromothripsis, we simulated data across 18 parameter settings, with 50 repeats for each parameter setting (Fig. 3b-e).<br>For validation of parameter inference, we simulated data across 18 parameter settings, with 10 repeats for each parameter setting (Fig. 5b-f).<br>To show the robustness of parameter inference, we also simulated data using breakpoints sampled from another patient with 10 repeats for two parameter settings (Supplementary Fig. 23).
Randomization	No randomisation was performed, because this is a descriptive study.
Blinding	No blinding was performed, because this is a descriptive study.

Reporting for specific materials, systems and methods

We require information from authors about some types of materials, experimental systems and methods used in many studies. Here, indicate whether each material, system or method listed is relevant to your study. If you are not sure if a list item applies to your research, read the appropriate section before selecting a response.

Materials & experimental systems

n/a	Involved in the study
☒	☐ Antibodies
☒	☐ Eukaryotic cell lines
☒	☐ Palaeontology and archaeology
☒	☐ Animals and other organisms
☒	☐ Clinical data
☒	☐ Dual use research of concern
☒	☐ Plants

Methods

n/a	Involved in the study
☒	☐ ChIP-seq
☒	☐ Flow cytometry
☒	☐ MRI-based neuroimaging

Plants

Seed stocks	No seed stocks were used, because this study does not involve plants.
Novel plant genotypes	No novel plant genotypes were used, because this study does not involve plants.
Authentication	No authentication was performed, because this study does not involve plants.
