# Supplementary material for: Cell-cycle dependent DNA repair and replication unifies patterns of chromosome instability
Source: Nat Commun. 2025 Mar 28;16:3033. doi: 10.1038/s41467-025-58245-z (PMC11953314; doi:10.1038/s41467-025-58245-z)
Supplement: Supplementary file 3 — Description of Additional Supplementary Files [file 41467_2025_58245_MOESM3_ESM.pdf]

File Name: Supplementary Data 1

Description: Summary of the posterior distributions of inferred parameters from simulated data presented in Fig. 5.

File Name: Supplementary Data 2

Description: Details of the single-cell whole-genome sequencing data used for the inferences in Fig. 6.

File Name: Supplementary Data 3

Description: Details of the bulk whole-genome sequencing data used for the inferences in Fig. 7.

File Name: Supplementary Data 4

Description: Structural variant (SV) signatures computed by SigProfiler from 1,815 Pan-Cancer Analysis of Whole Genomes (PCAWG) samples with structural variants, among which SV2, SV4, SV6, and SV8 correspond to the Catalogue of Somatic Mutations in Cancer (COSMIC) signatures.
